# Identifying spatio-temporal seizure propagation patterns in epilepsy using Bayesian inference

**DOI:** 10.1038/s42003-021-02751-5

**Published:** 2021-11-01

**Authors:** Anirudh N. Vattikonda, Meysam Hashemi, Viktor Sip, Marmaduke M. Woodman, Fabrice Bartolomei, Viktor K. Jirsa

**Affiliations:** 1 Aix Marseille Univ, INSERM, INS, Institut de Neurosciences des Systèmes, Marseille, France; 2 Epileptology Department and Clinical Neurophysiology Department, Assistance publique des Hopitaux de Marseille, Marseille, France

**Keywords:** Epilepsy, Dynamical systems, Machine learning

## Abstract

Focal drug resistant epilepsy is a neurological disorder characterized by seizures caused by abnormal activity originating in one or more regions together called as epileptogenic zone. Treatment for such patients involves surgical resection of affected regions. Epileptogenic zone is typically identified using stereotactic EEG recordings from the electrodes implanted into the patient’s brain. Identifying the epileptogenic zone is a challenging problem due to the spatial sparsity of electrode implantation. We propose a probabilistic hierarchical model of seizure propagation patterns, based on a phenomenological model of seizure dynamics called Epileptor. Using Bayesian inference, the Epileptor model is optimized to build patient specific virtual models that best fit to the log power of intracranial recordings. First, accuracy of the model predictions and identifiability of the model are investigated using synthetic data. Then, model predictions are evaluated against a retrospective patient cohort of 25 patients with varying surgical outcomes. In the patients who are seizure free after surgery, model predictions showed good match with the clinical hypothesis. In patients where surgery failed to achieve seizure freedom model predictions showed a strong mismatch. Our results demonstrate that proposed probabilistic model could be a valuable tool to aid the clinicians in identifying the seizure focus.

## Abstract (web summary)

Vattikonda et al. combine a conceptual mathematical model of seizure generation with personalized information about brain connectivity to tackle the challenge of predictive brain surgery. The validated model was used to successfully determine the outcome of surgery in retrospective patient data for removal of epileptogenic brain tissue.

## Introduction

Epilepsy is a common neurological disorder, characterized by seizures, affecting more than 50 million people worldwide^1^. Drug-resistant epilepsy is a class of epilepsy where medication fails to control seizures and is observed in nearly 25% of epilepsy patients. In such cases, clinical treatment usually involves surgical resection of brain regions that are considered to be originating seizures. Success rates of epilepsy surgery, ranging between 50% and 60%, prescribe a need for developing better methods to identify an epileptogenic zone (EZ). In this work, we propose a Bayesian framework based on a dynamical model of epileptic seizures, namely Epileptor^2,3^, for identifying spatio-temporal seizure propagation patterns.

Focal epileptic seizures are characterized by seizures originating in one or more regions, generally referred to as EZ, and propagating to other regions that are connected to regions in the EZ, generally referred to as a propagation zone (PZ). Such seizure propagation patterns can be adequately described given the seizure-onset and -offset times of all the regions recruited by the seizure. In focal epilepsy, a transient change in signal power is a characteristic feature of seizure onset and offset. Stereotactic electroencephalography (SEEG) log power computed over a sliding window captures such transients in SEEG signal power reliably. In the previous work^4,5^, patient-specific information such as anatomical connectivity obtained from non-invasive imaging techniques is combined with the dynamical models of local neuronal activity (such as Epileptor) to describe the individual’s spatio-temporal brain activity at the macroscopic scale. These studies demonstrated that a network of coupled Epileptors can predict various patient-specific seizure propagation patterns given that EZ is known. Hence, we hypothesize that by inverting the coupled Epileptor model to best fit the SEEG log power, it would be possible to build patient-specific virtual models of spatio-temporal seizure propagation patterns. However, such an inversion is non-trivial primarily due to the following: (a) large dimensionality of the parameter space, which includes unknown model parameters and the unobserved source states; (b) spatial sparsity of SEEG measurements; and (c) source mixing at the sensors, i.e., the activity recorded by the SEEG sensors could be a mixture of activity from different brain regions in the neighborhood of the sensor.

In order to address these issues, in this work, we use Bayesian inference paradigm to perform model inversion. Bayesian inference offers a flexible framework for incorporating any prior knowledge such as plausible range of model parameters, dynamics of unobserved brain states, and prior hypothesis on the seizure focus. These priors constrain the parameter space, thus enabling efficient exploration of posterior distribution of parameters. In the field of neuroscience, Bayesian inference has been extensively and fruitfully used for model inversion by a class of models called dynamic causal modeling (DCM)^6^. DCM is a Bayesian framework for inferring physiological mechanisms that could generate observations obtained from various neuroimaging techniques such as functional magnetic resonance imaging (MRI) and electroencephalography (EEG)^7,8^. Using DCM for cross-spectral density, Papadopoulou et al.^9^ have inferred modulations in synaptic efficacy of intrinsic and extrinsic connections within and between two regions during seizure onset. They have demonstrated that DCM provides a mechanistic insight into the underlying seizure generation and propagation processes provided a good estimate of the EZ network is available. Although variants of DCM such as spectral DCM^10^ and regression DCM^11^ are shown to scale to whole-brain network models, of up to 66 regions, inference using DCM for a whole-brain model is not yet demonstrated in the context of epilepsy. Recently, efficient and robust inversion of seizure propagation on whole-brain scale was achieved by simplifying the seizure dynamics using a threshold model^12^; however, this approach considerably restricts the range of model dynamics. In this study, we demonstrate inversion of a phenomenological model of epileptic seizure dynamics at the whole-brain level, containing 164 regions, with the objective of identifying the EZ.

Taking advantage of recent advances in probabilistic programming languages (PPLs) for Bayesian inference, such as Stan^13^, we demonstrate that it is possible to invert the coupled Epileptor model that best explain each patient’s intracranial recordings. The problem of model inversion is framed as an optimization problem by defining an objective function over Epileptor parameter space such that the maxima of this function correspond to the parameters that best explain the SEEG data. Specifically, a joint probability density is defined over the Epileptor parameters and observed log power of intracranial recordings by embedding Epileptor equations as priors on brain source dynamics. Next, using maximum a posteriori (MAP) techniques, Epileptor parameters are optimized to best fit intracranial data. In order to test the validity of this approach, first the accuracy of model predictions is tested against the synthetic data generated using The Virtual Brain (TVB)^14^. By fitting the synthetic data, we show that the optimized model can accurately identify the spatio-temporal seizure propagation patterns. Finally, patient-specific virtual epileptic patient (VEP), models are built for a retrospective patient cohort containing 25 patients with varying surgery outcome. As the empirical data set does not contain the whole-brain source activity, model predictions are validated against the clinical EZ hypothesis. In patients who are seizure-free after surgery, we demonstrate that optimized VEP models are able to identify the EZ. More interestingly, in patients who are not seizure-free, we found that the model predictions do not match with the clinical hypothesis of EZ. These results suggest that the proposed approach can be a valuable tool for clinicians in identifying EZ to improve outcomes of epilepsy surgery.

## Results

**Fig. 1:**
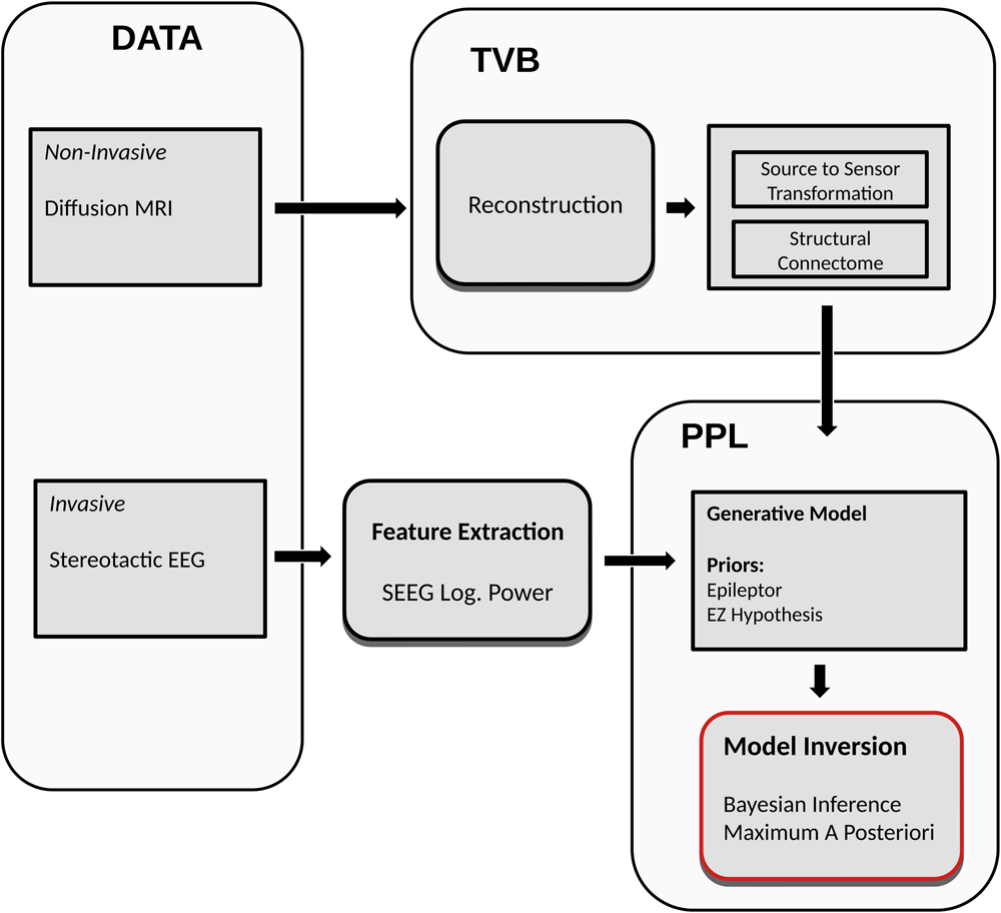
End-to-end workflow for identifying spatio-temporal seizure propagation patterns in focal drug-resistant epilepsy patients. Diffusion MRI and SEEG data are collected for each patient. From this data, structural connectome and the transformation from source-to-sensor space is computed. SEEG recordings are preprocessed to extract data features such as log power over a sliding window, which could be modeled using 2D Epileptor. A generative model is defined over the 2D Epileptor parameters and observed SEEG log power using a PPL called Stan. Model inversion is performed using maximum a posterior techniques to identify 2D Epileptor parameters that best explain the observations. PPL, probabilistic programming language; SEEG, stereotactic electroencephalography; TVB, The Virtual Brain.

The workflow for identifying seizure propagation pattern (Fig. 1) briefly consists of the following steps: (a) estimating structural connectome (SC) and source to sensor space transformation from diffusion MRI data and electrode implantation, respectively; (b) extracting data features, log power over a sliding window, from the observed SEEG data; (c) defining a generative model that describes the joint probability density over the observations and Epileptor model parameters; (d) performing model inversion using MAP techniques in order to infer the model parameters and latent source states that best fit to the observed SEEG log power; (e) computing the seizure-onset times of all regions recruited by the seizure by thresholding the inferred latent states; and (f) identifying the EZ and PZ: all regions with onset times between the lowest onset time and a small onset tolerance window are classified as part of EZ, and regions with onset times greater than this window are classified as part of PZ. See “Methods” for a more detailed description of each of these steps.

### Model validation against synthetic data

**Fig. 2:**
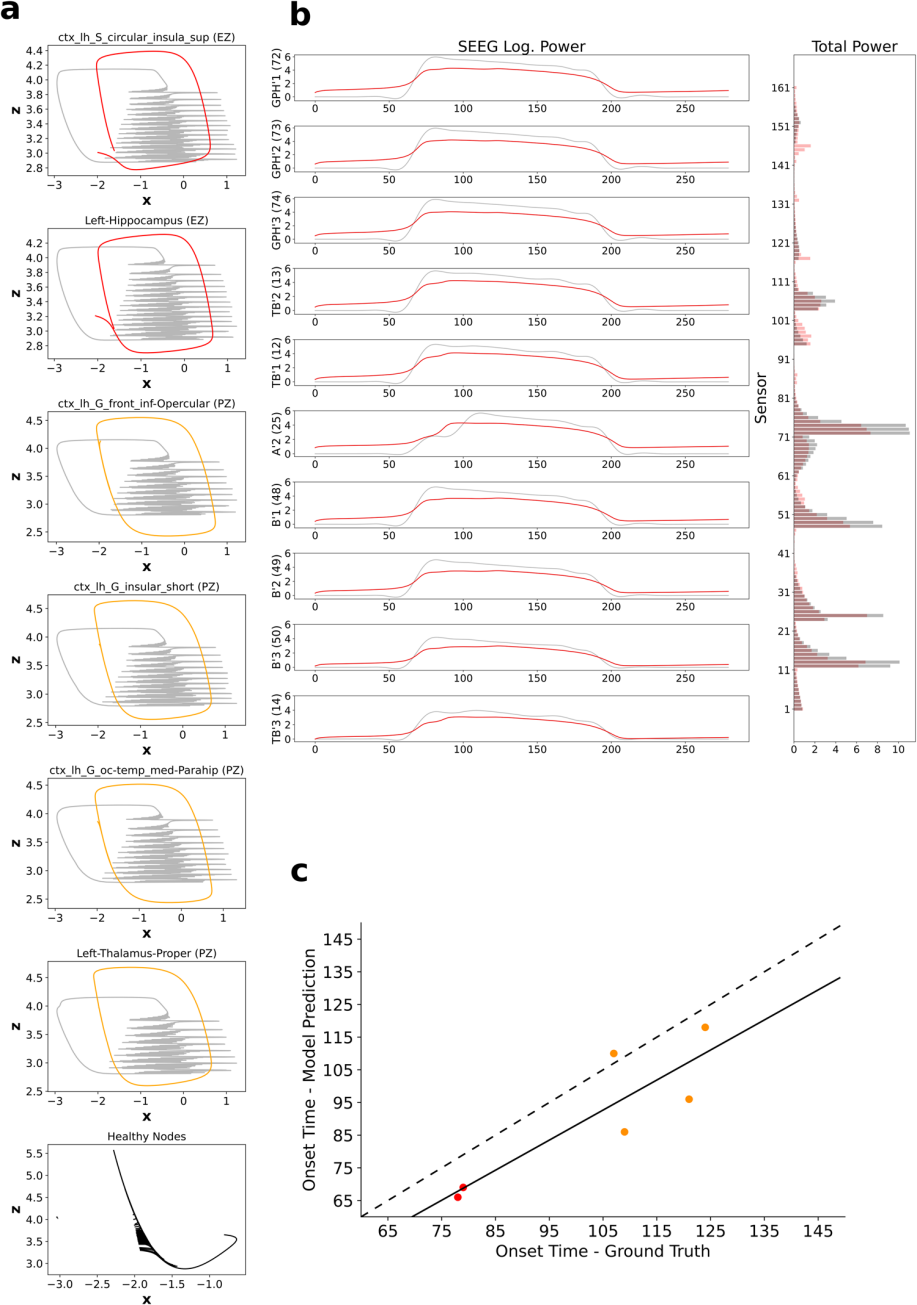
Inferred seizure propagation pattern in synthetic data. **a** Phase plane plots of inferred 2D Epileptor source dynamics. For comparison with the ground truth, generated using 5D Epileptor, time series of local field potential (*x*_1_(*t*) + *x*_2_(*t*)) and the corresponding permittivity variable *z* is shown in gray (see “Synthetic data” in “Methods”). Inferred source dynamics of regions in EZ is shown in red, regions in PZ is shown in orange, and the remaining regions are shown in black. **b** Comparison of model-predicted SEEG log. Power (left) of ten sensors with highest power and the augmented data feature: total sensor power (right). Observations are shown in gray and model predictions are shown in red. **c** Seizure-onset times in ground truth vs. model predictions. Red dots represent the seizure-onset times of regions in EZ and orange dots represent the seizure-onset times of regions in PZ. Solid line represents the linear regression fit and dashed line represents a perfect fit. EZ, epileptogenic zone; PZ, propagation zone.

To test the accuracy and identifiability of the proposed generative model in inferring the spatio-temporal seizure propagation patterns, first it is tested against a synthetic data set (see  “Synthetic data” in “Methods”), so that the inferred seizure propagation pattern can be validated against the ground truth at the source level. Inferred seizure propagation obtained by maximizing over the posterior density (Eq. (5)) of the synthetic data is shown in Fig. 2. State space dynamics of the simulated and inferred source activity are shown in Fig. 2a. All the six regions recruited by the seizure, with two regions in EZ and four regions in PZ, in the ground truth are accurately inferred to be recruited by seizure in the inferred source activity. Figure 2b shows the fit between the observed and predicted SEEG log power. The seizure propagation pattern is then identified by computing the onset times of all the regions that are recruited in the seizure. Seizure-onset times, computed by thresholding the source activity, in the ground truth and model predictions are shown in Fig. 2c. Both the regions in EZ are inferred accurately to have an earlier onset time relative to the onset times of regions in PZ and no other regions outside EZ and PZ are inferred to be recruited by the seizure.

**Fig. 3:**
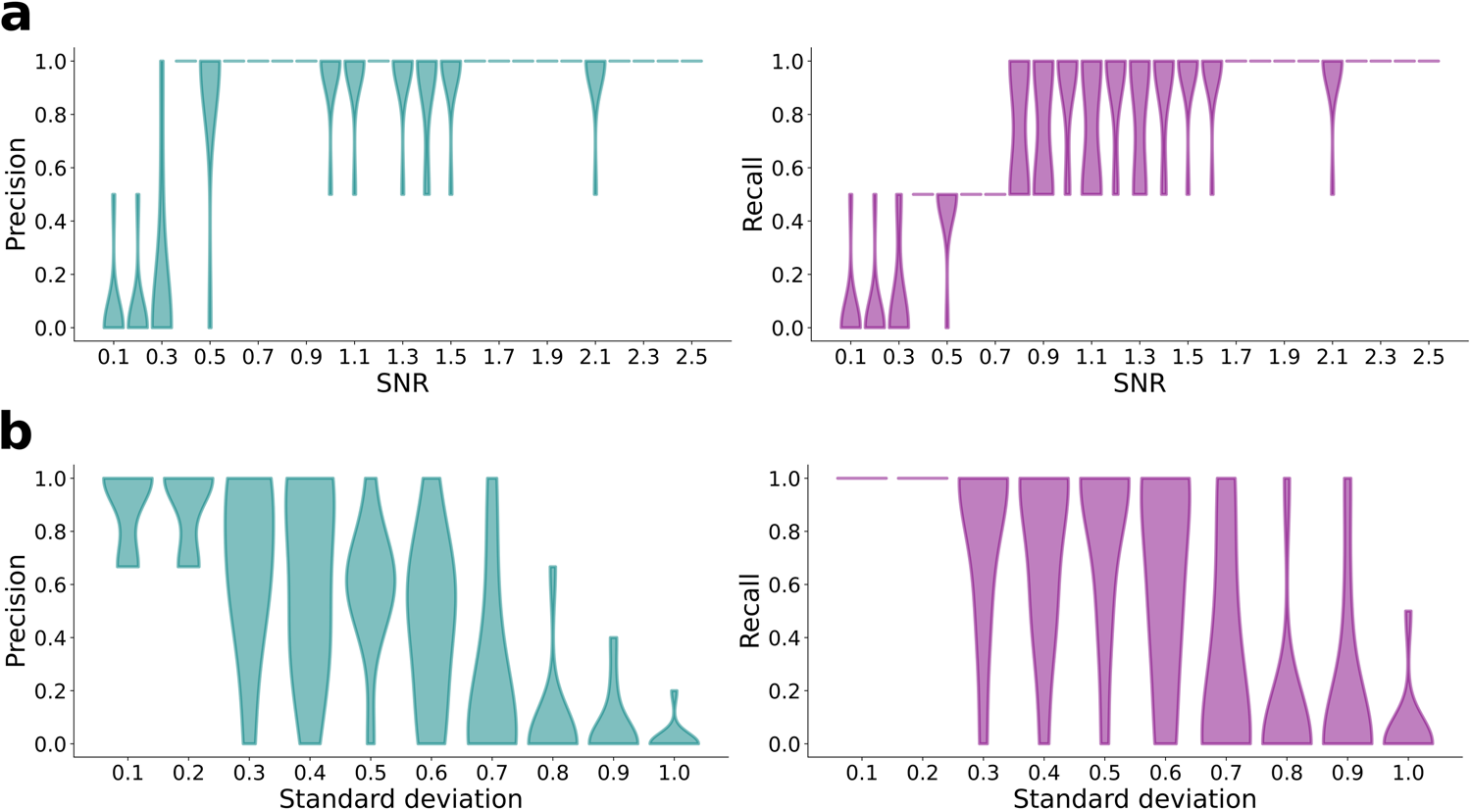
Analysis of robustness of MAP estimation of epileptogenic zone. **a** Accuracy of the estimated EZ as the signal-to-noise ratio is increased from 0.1 to 2.5. For each SNR, *n* = 50 independent data sets are generated and EZ is estimated using MAP. **b** Accuracy of estimated EZ with MAP initialized at different values of parameters. Initial conditions are generated using a proposal distribution, which is defined such that it has the same mean as the priors of the parameters but with SD ranging from 0.1 to 1.0. At each SD, *n* = 50 independent samples are generated and used as initial conditions for MAP. EZ, epileptogenic zone; MAP, maximum a posteriori; SNR, signal-to-noise ratio.

Robustness of the inference is then tested against a range of scenarios such as the signal-to-noise ratio (SNR) in the observations, different initialization of the MAP optimization, and different number of regions in EZ and PZ. In order to test the sensitivity of the inference against different levels of noise in the observations, Gaussian noise with zero mean and varying SD is added to the simulated SEEG data, to generate different data sets with SNR, averaged across SEEG channels, ranging from 0.1 to 2.5. For each SNR, 50 data sets are generated and EZ is inferred using MAP. Precision and recall of the inferred EZ for each SNR is shown in Fig. 3a. We found that inference is able to accurately identify the EZ for SNR values > 0.9. As MAP is an optimization procedure, it could lead to different results if the objective function, here the posterior density over model parameters, is multimodal or non-convex. Typically, if the objective function is multimodal, MAP is initialized randomly at different regions of the parameter space and the estimate with highest posterior probability is chosen as the best estimate. In the inference procedure proposed in this study, evaluating the objective function at any point involves numerical integration of the two-dimensional (2D) Epileptor model (Eq. (8)). This has prohibited us from using a completely random initialization (i.e., initial conditions generated from a uniform distribution) of MAP as the numerical integration diverges in some regions of parameter space leading to numerical errors in evaluating the posterior probability. To avoid such divergences while using a multi-start procedure with MAP, we have constrained the initial conditions to well-behaved regions of the parameter space. In order to achieve this, initial conditions are sampled from ten different proposal distributions, which are defined to have the same mean as priors but with SD ranging from 0.1 to 1.0. As the prior distributions are defined taking into account the dynamical properties of the 2D Epileptor model, sampling from such a proposal distribution ensures that the initial conditions are within the well-behaved regions of the parameter space. For each of the 10 proposal distributions, 50 samples are generated and MAP is performed using those samples as the initial values of the parameters. Accuracy of the inferred EZ for each proposal distribution is shown in Fig. 3b. Inference is able to accurately identify EZ when SD of the proposal distribution is <0.7 but gets stuck in a local minimum for larger SDs. In high-dimensional parameter spaces, as the SD of the proposal distribution increases, the probability that the sampled initial conditions are close to the mean decays exponentially. Hence, for proposal distributions with larger SD, it would require more samples of initial conditions to get performance similar to that of proposal distributions with low SDs. Similar results are obtained in three other synthetic data sets with various number of regions in EZ and PZ, and shown in Supplementary Figs. 1 and 2. As best performance is achieved for low SDs of the proposal distribution, we have used the mean of priors as initial conditions, while performing MAP on empirical data.

### Model validation against empirical data

Next, the model is tested against a retrospective patient cohort of 25 drug-resistant epilepsy patients who underwent surgery. The cohort is divided into two groups based on the outcome the of surgery as follows: (i) Engel score I and II: patients who are either seizure-free or show rare disabling seizures and (ii) Engel score III and IV: patients with minimal or no worthwhile improvement. For each group, patient-specific models are generated by inverting 2D Epileptor model (Eq. (6)) against each patient’s SEEG data.

**Fig. 4:**
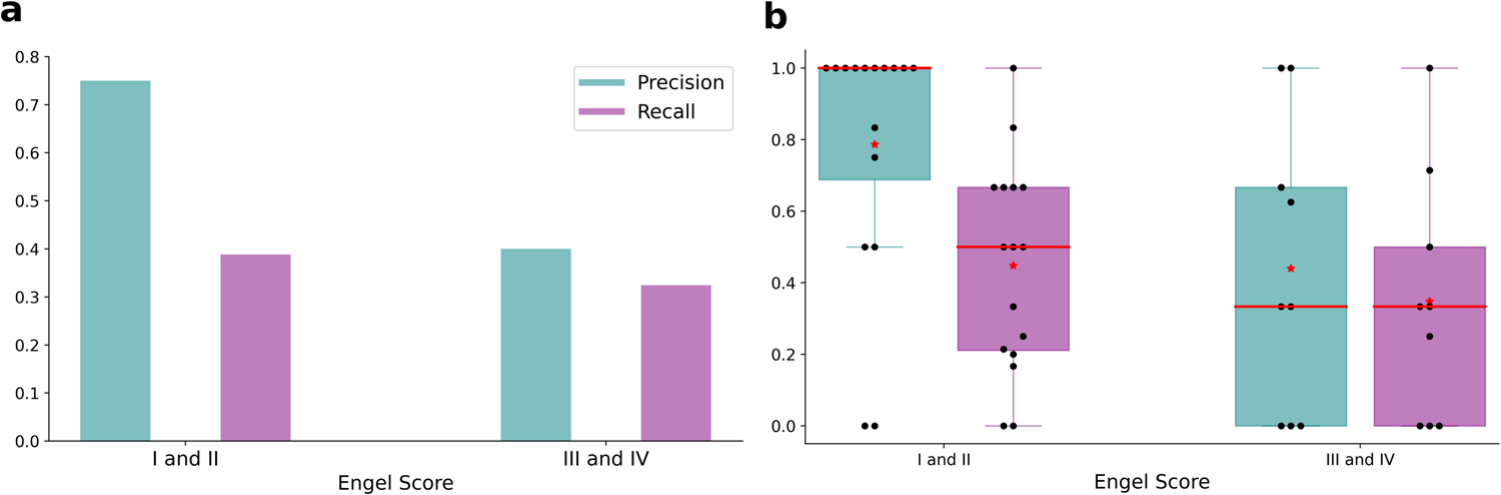
Precision and recall of model-predicted EZ compared to clinical EZ hypothesis. **a** Precision/recall across all patients in the two patient groups: Engel score I and II: patients with post-surgical seizure freedom or with rare disabling seizures (*n* = 16); Engel score III and IV: patients with minimal or no worthwhile improvement (*n* = 9). **b** Distribution of precision/recall, computed per patient, in each group. In these box plots, red lines represent the median, red stars represent mean, and the data are overlaid as black circles. In both (**a** and **b**), onset tolerance (*t*_*ϵ*_) of 10 s is used for identifying EZ from the inferred source activity. The whiskers are located at 1.5 times the interquartile range from above and below. EZ, epileptogenic zone.

**Fig. 5:**
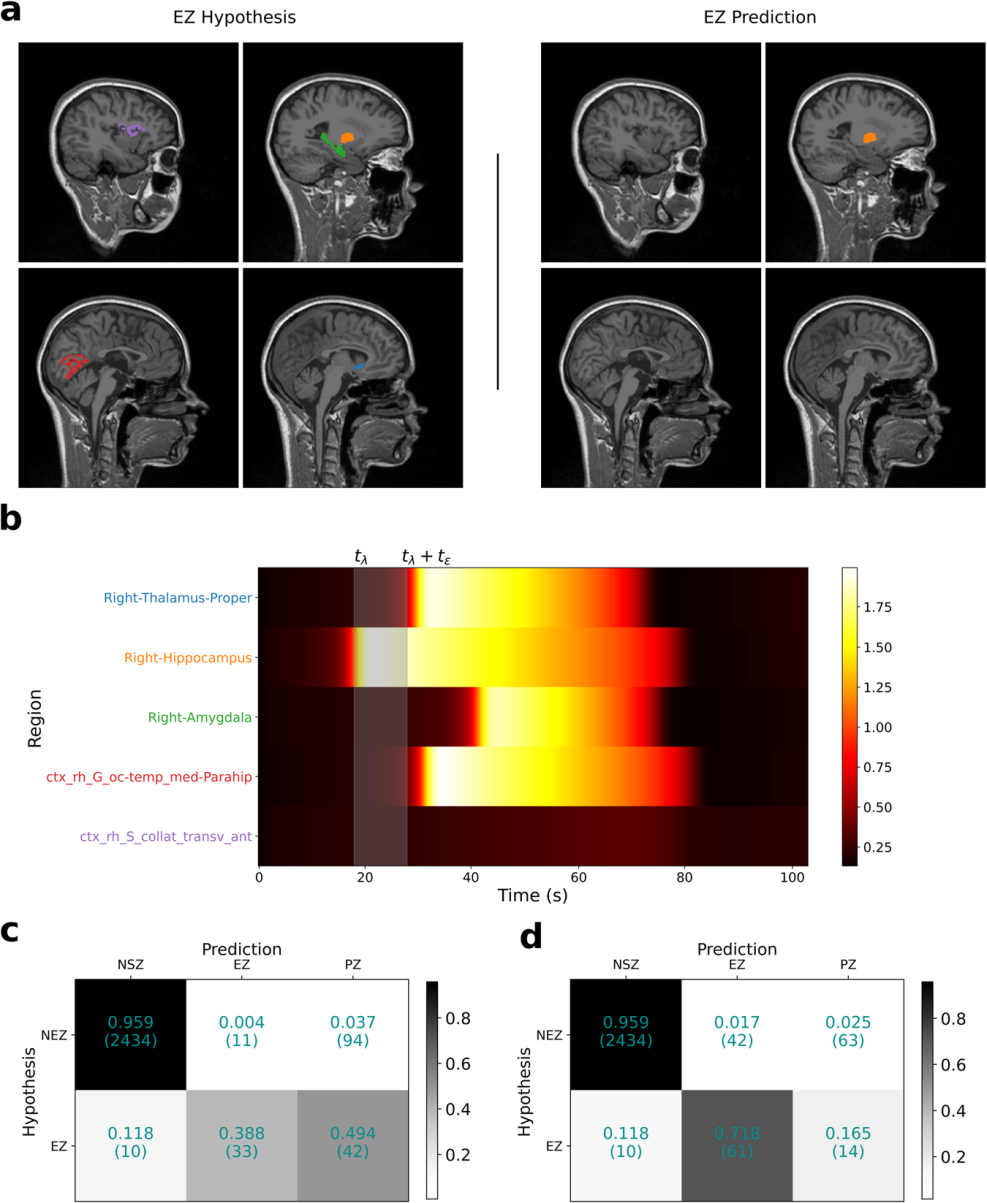
Inferred seizure propagation pattern in patient LMA with Engel score I. **a** Comparison of clinical EZ hypothesis (left) with model-predicted EZ (right). **b** Model-predicted seizure propagation pattern as observed in the predicted source power. **c** Confusion matrix comparing clinical classification of brain regions and model prediction-based classification across all patients in the Engel score I/II group at onset tolerance (*t*_*ϵ*_) of 10 s. **d** Same as in (**c**), except *t*_*ϵ*_ = 30 s. Fitting between model predictions and observed data features is shown in Supplementary Fig. 3. EZ, epileptogenic zone.

Precision and recall of the model-predicted EZ compared to the clinical EZ hypothesis across all patients in each group is shown in Fig. 4a. As precision across all patients in a group can potentially be biased when the number of regions in EZ are not uniformly distributed, we also computed precision/recall per patient. The distribution of precision/recall computed per patient in each group is shown in Fig. 4b. In the group with Engel score I and II patients, model-predicted EZ showed a precision of 0.75 and a recall of 0.38 at an onset tolerance threshold (*t*_*ϵ*_) of 10 s. It is noteworthy that the low recall does not imply that 60% of regions in EZ clinical hypothesis are not inferred as seizing, because some of the regions in the EZ hypothesis, although inferred to be seizing, are classified as part of the PZ. To illustrate this, the inferred seizure propagation pattern of a patient with Engel score I is shown in Fig. 5. Out of the five brain regions in the clinical EZ hypothesis, the model predicted four regions to be recruited by the seizure with one subcortical region (right hippocampus) in EZ and three regions (right thalamus proper, right amygdala, and ctx-rh-G-oc-temp-med-Parahip) as part of PZ. To quantify this at the group level, we calculated the confusion matrix between the clinical hypothesis-based classification and the model prediction-based classification of the regions. The clinical classification is a binary classification labeling each region as part of EZ or not part of EZ, whereas the model prediction-based classification consists of three classes: (i) regions that are part of EZ, (ii) regions in PZ, and (iii) regions that are not recruited by the seizure. At an onset tolerance of 10 s, 88.2% of regions in clinical EZ hypothesis are predicted to be recruited by the seizure, with 38.8% in EZ and 49.4% in PZ (Fig. 5c). As the onset tolerance is increased to 30 s, the recall increased from 0.38 to 0.71 (Fig. 5d).

**Fig. 6:**
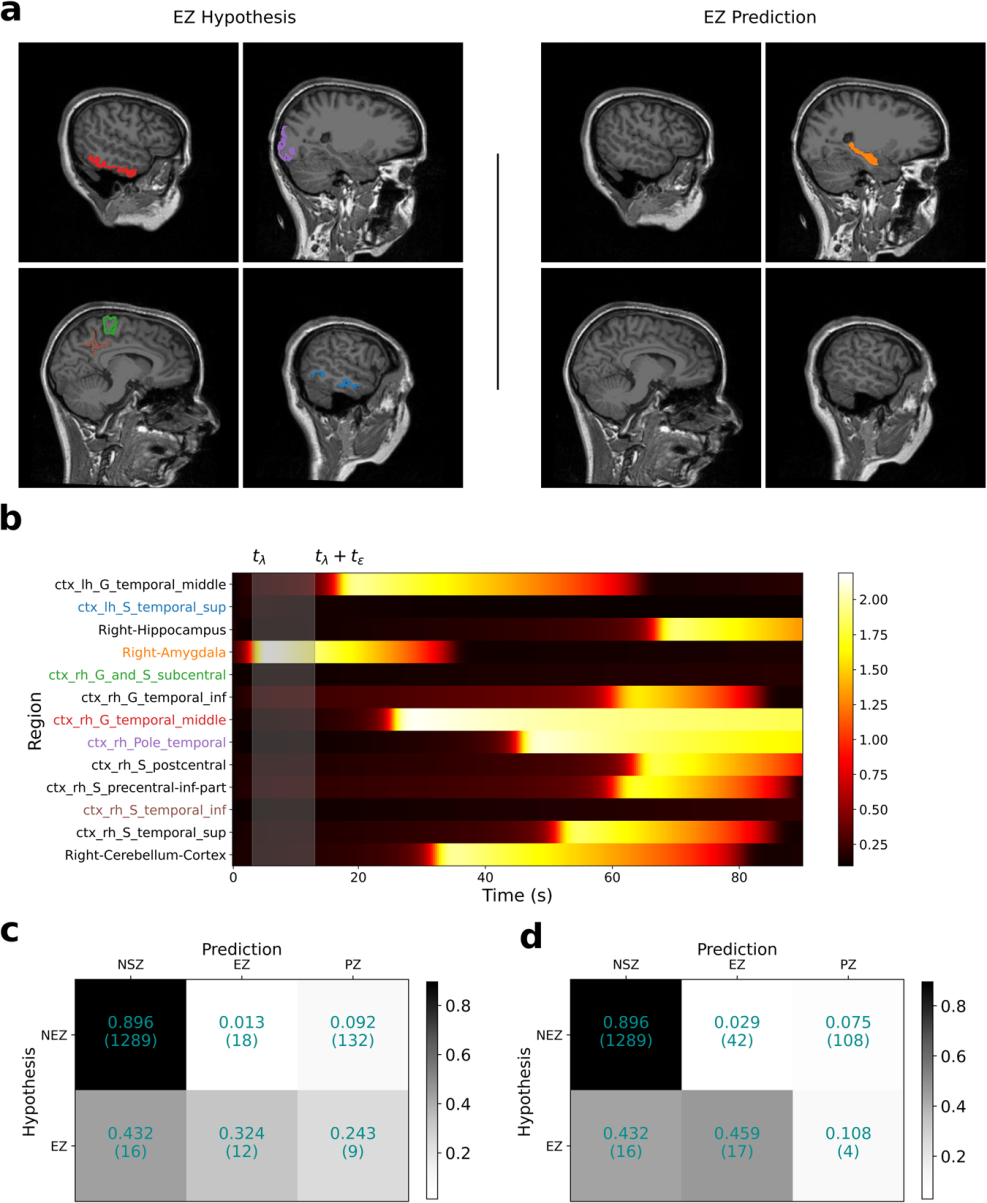
Inferred seizure propagation pattern in patient FC with Engel score IV. **a** Comparison of clinical EZ hypothesis (left) with model-predicted EZ (right). **b** Model-predicted seizure propagation pattern as observed in the predicted source power. **c** Confusion matrix comparing clinical classification of brain regions and model prediction-based classification across all patients in the Engel score III/IV group at onset tolerance (*t*_*ϵ*_) of 10 s. **d** Same as in (**c**), except *t*_*ϵ*_ = 30 s. Fitting between model predictions and observed data features is shown in Supplementary Fig. 4. Ez, epileptogenic zone.

In the second group, with Engel score III and IV patients, inferred EZ showed a strong mismatch with clinical hypothesis in more than half of the patients. For this group, model predictions showed a precision of 0.40 and a recall of 0.32. The low precision and recall imply that most of the regions in the clinical hypothesis are predicted to be either as not recruited by the seizure or as a part of the PZ. In addition, regions not part of the clinical EZ hypothesis are inferred to be part of EZ. For illustration, comparison of the clinical EZ hypothesis and model-predicted EZ for a patient with Engel score IV is shown in Fig. 6a. In this patient, none of the five regions in clinical hypothesis are inferred as part of EZ, but rather a subcortical region outside EZ hypothesis (right amygdala) is inferred as the seizure focus. Two regions in the temporal lobe that are part of clinical hypothesis (ctx-rh-G-temporal-middle and ctx-rh-pole-temporal) are inferred to be recruited later by the seizure as part of PZ (Fig. 6b). As shown in the confusion matrix for this group in Fig. 6c, at an onset tolerance of 10 s, 67.5% of the regions in clinical EZ hypothesis are inferred to be outside EZ with 24.3% inferred as part of PZ and 43.2% of regions as not recruited. Unlike the first group, even at an onset tolerance of 30 s (Fig. 6d), 54% of regions are still inferred to be outside EZ. However, it is noteworthy that as shown in Fig. 4b, two out of the nine patients in this group showed a good match with the clinical EZ hypothesis. We believe this could be happening as: (1) the hypotheses about the EZ is correct but during the surgery not enough tissue is removed from those regions; (2) the hypotheses is wrong but the data are not informative enough, potentially due to the implantation being far from the true EZ, in which case the priors dominate the likelihood, leading to a posterior mode around the wrong hypothesis.

**Fig. 7:**
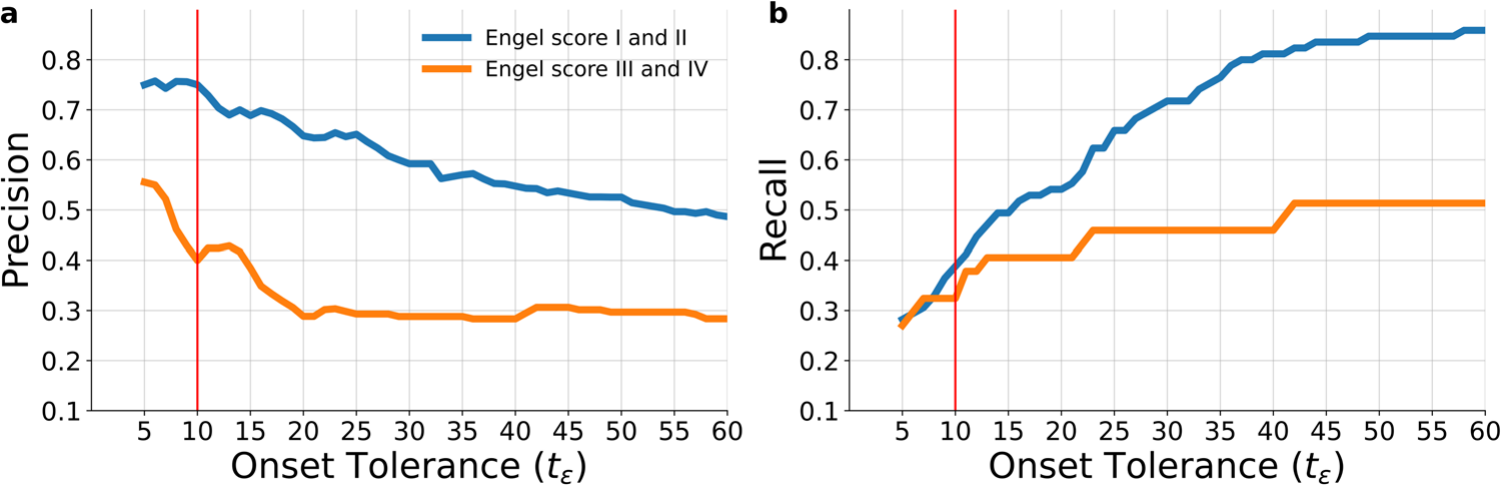
Precision and recall across various onset tolerance thresholds. **a** Precision of both the patient groups as the onset tolerance threshold *t*_*ϵ*_ is varied from 1 to 60 s. **b** Recall of both the patient groups as the onset tolerance threshold *t*_*ϵ*_ is varied from 1 to 60 s. The red vertical line represents the onset tolerance used in previous results of this study.

To investigate the effect of onset tolerance threshold on model prediction, precision and recall are calculated as the threshold (*t*_*ϵ*_) is increased from 1 to 60 s for both the patient groups (Fig. 7). Across all thresholds, precision in the Engel I/II group is higher than precision in the Engel III/IV group. The number of false positives have substantially increased in Engel III/IV group for thresholds beyond 5 s as seen by the dip in precision. The recall in both groups is similar for thresholds <10 s, as some of the regions in clinical hypothesis are classified as PZ, as shown in Figs. 5 and 6. However, as the onset tolerance is increased, the recall in Engel III/IV converged to 0.51, implying that ~50% of regions in clinical hypothesis are not inferred to be recruited by the seizure.

### Prediction accuracy with bad EZ hypothesis

**Fig. 8:**
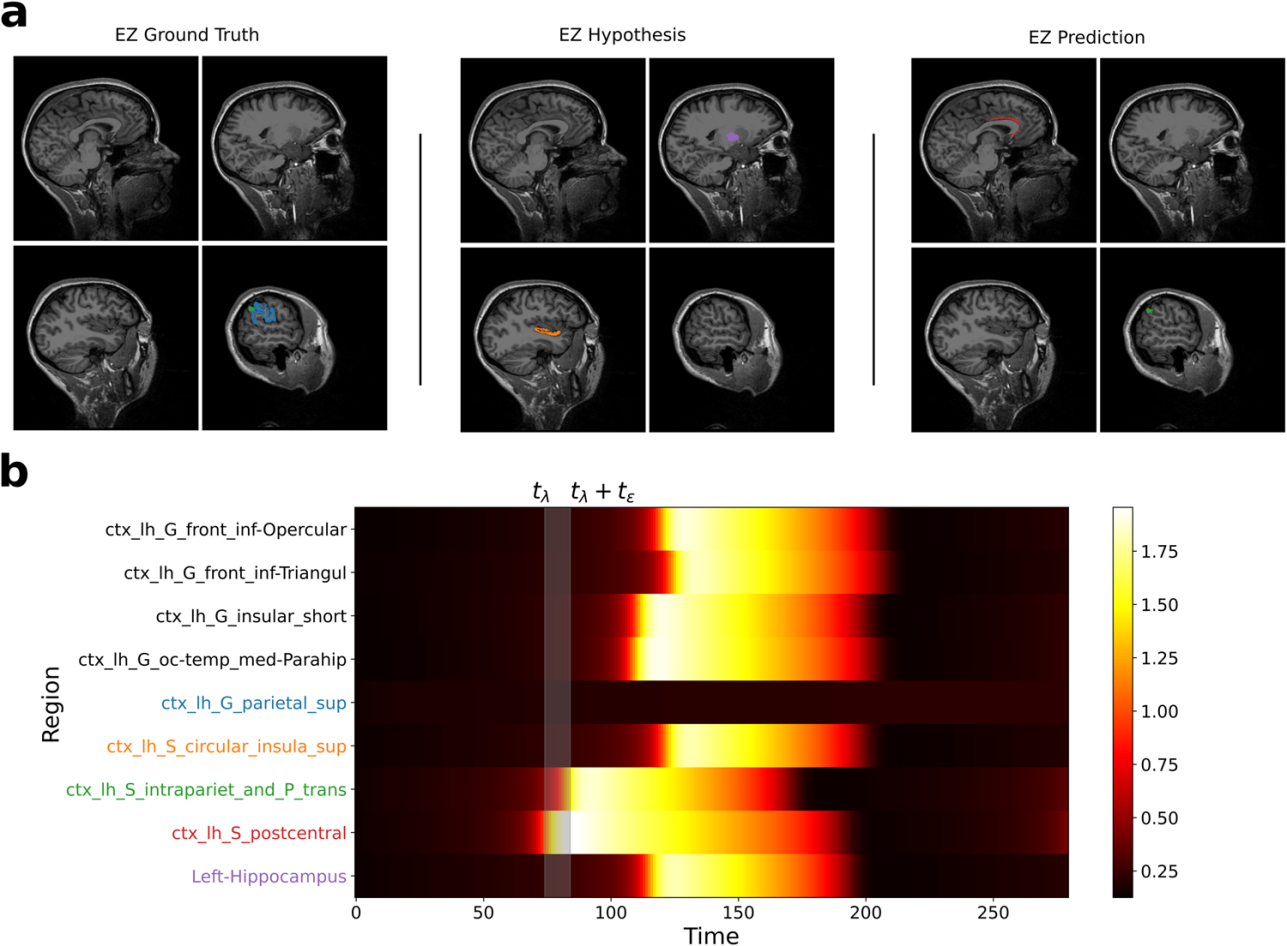
Inferred seizure propagation in synthetic data with bad EZ hypothesis. **a** Comparison of regions in epileptogenic zone from ground truth, hypothesis, and model prediction. **b** Model-predicted seizure propagation pattern. EZ, epileptogenic zone.

In Fig. 2, using synthetic data we have shown that model predictions are accurate when the EZ hypothesis is good. In concordance with this, in empirical data, model predictions in seizure-free group, i.e., Engel I/II have shown a good match with clinical EZ hypothesis. However, in the Engel III/IV group, model predictions have shown a strong mismatch with EZ hypothesis. In order to understand how to interpret the model predictions when the EZ hypothesis is bad, we have tested the accuracy of model predictions in synthetic data (Supplementary Fig. 5) with a bad EZ hypothesis. Figure 8a shows a comparison of model-predicted EZ with the ground truth and the bad hypothesis. We found the results to be similar to the results in Engel IV patient in Fig. 6. Model-predicted EZ did not include any of the regions from the wrong hypothesis but includes one of the regions from the ground truth and one false positive.

## Discussion

Focal drug-resistant epilepsy is characterized by seizures originating in one or more regions of the brain and quickly propagating to other regions. Identifying such spatio-temporal seizure propagation patterns has been a challenging problem due to the spatial sparsity of electrode implantation. In this study, we propose a hierarchical probabilistic model of seizure propagation based on a phenomenological model of seizure onset and propagation called Epileptor^2,3^. The full Epileptor model and its associated dynamotypes describe a large repertoire of dynamic phenomena^15^, capturing the full range of bifurcation types (for onset and offset), amplitude, and frequency scaling, multistability of ictal and non-ictal phases, as well as slow-variable fluctuations. Taking a Bayesian paradigm, using prior knowledge about a patient’s anatomical information, specifically structural network and clinical estimation of EZ, and the likelihood of observing specific propagation patterns, a posterior probability density is defined for each patient. We demonstrated that using Bayesian inference and state-of-the-art MAP techniques^13,16^, personalized models of patient-specific seizure propagation patterns can be built by optimizing over the posterior probability density, i.e., inverting the Epileptor model. For the model inversion, model reduction was guided by criteria rendering the approach usable in application, which meant, in this case, that we emphasized the power envelope function, thereby losing the data features on the fast timescales (bifurcation type for instance) and prioritizing the network features (effects of connectivity and propagation). This reduction has been justified by the dominance of one seizure type, which is well represented by the power envelope and by the network nature of seizure propagation^17^.

The accuracy of estimated model is validated by comparing the model predictions against synthetic data, where the ground truth is available. We have shown that the estimated model is able to accurately identify the EZ (Figs. 2 and 3). Next, the model accuracy is validated against a retrospective patient cohort, containing 25 patients. Although model validation in synthetic data is straightforward, as we know the exact ground truth, it is not trivial in empirical data. The state-of-the-art in validating model predictions in focal epilepsy patients is to compare the estimated EZ with the clinical hypothesis of EZ used for surgery^12,18–20^. Following the same approach, we have shown that model predictions match well (Fig. 4) with the clinical hypothesis in patients where surgery resulted in seizure freedom, whereas if different from the clinical hypothesis, surgery tended to fail, to achieve seizure freedom. These results on a small retrospective data set of 25 patients provide face validity for the proposed approach and the estimated personalized models, laying the groundwork for systematic testing in larger prospective data sets in clinical trial. When making the link of the comparison reliability for synthetic data to the one of real world data, there are numerous issues regarding the ecological validity of the entire approach of EZ such as the network nature of the epileptic brain (as opposed to a spatially continuous neural field), the stationary organization of the EZ’s activity (as opposed to traveling waves of ictal discharges), or, beyond the EZ, the strong parcellation dependence of the electric forward solution mapping source-to-sensor space. All of these issues are essentially non-existent in the synthetic data by construction but they are a major contaminant factor detrimental to the model inversion process for empirical data, however, beyond the scope of this study. The target level of explanatory power in this study can only be the current state of the art, which we have shown to be well supported by the performance metrics provided in this study.

In the proposed hierarchical model, the lowest level consists of the Epileptor model parameters, which, through a nonlinear transformation, determine the second level of parameters, i.e., latent states of source dynamics. These source dynamics then determine the fit to the observed SEEG log power by using a linear transformation from source-to-sensor space. There are two levels of degeneracy in this hierarchy: (1) different realizations of Epileptor parameters could lead to similar source dynamics due to the structural non-identifiability^21,22^ in the transformation from Epileptor parameters to latent states and (2) different realizations of latent states could lead to similar dynamics in sensor space, as the transformation matrix from source-to-sensor space is singular. The second degeneracy is addressed by constraining the latent state transitions to follow the dynamics as governed by Epileptor equations (Eq. (8)). The first level of degeneracy requires a reparametrization of the Epileptor model parameters and is not explored in this study. However, the latent states are sufficient to identify the seizure propagation pattern. Moreover, without a reparametrization, understanding the seizure propagation from a specific parameter values of a coupled Epileptor model is not obvious unless accompanied by the corresponding source dynamics.

One of the main advantages of a Bayesian approach is that much of the relevant information about the seizure can be readily incorporated into the generative model using priors. Moreover, the priors can be adjusted to be strong or weak based on the confidence in the a priori knowledge. If the priors are supported by the observations, it is automatically reflected in the posterior density by an increased probability density in the parameter subspace supported by priors. Whereas, when the priors and the observations are not in agreement, the observation likelihood outweighs any weakly informative priors and the posterior shows a relatively higher density in the parameter subspace supported by likelihood. In this study, we have used strong priors on the source dynamics and weakly informative priors on excitability of the brain regions. As expected, in the case of Engel score I/II patients where the priors and observations are in agreement, the model predictions showed a good match with clinical EZ hypothesis (Fig. 5), whereas, for rest of the patients where the priors on excitability are not in agreement with the observations the model predictions showed a strong mismatch with the clinical hypothesis Fig. 6).

In this study, parameter inference is performed using MAP. Although MAP is computationally less expensive, it has several limitations. MAP is an optimization technique and the optimization may get stuck in a local optima when the posterior is non-convex. It is also possible that posterior is multimodal, i.e., different configurations of the model parameters can explain the data equally well, in which case point estimates from MAP would be insufficient even if one of the global optima is identified. Even when the posterior is unimodal, point estimates obtained from MAP may not be representative of the whole posterior, as MAP does not capture the uncertainty in parameters. Moreover, as the objective function optimized in the case of MAP is a probability density function, any reparameterization could change the posterior modes. These issues could possibly be addressed by sampling the whole posterior using Markov chain Monte Carlo (MCMC)-based algorithms, which provide a holistic view of the posterior instead of point estimates provided by MAP. However, even advanced gradient-based MCMC sampling algorithms such as Hamiltonian Monte Carlo (HMC), No U-Turn sampler (NUTS)^23,24^ fail to sample the posterior density efficiently when parameters in a high-dimensional hierarchical model show strong nonlinear correlations or if the posterior exhibits pathological geometries such as Neal’s funnel with varying curvature^25^. Unfortunately, the probabilistic model proposed here does exhibit these pathologies, making it computationally infeasible for sampling with HMC/NUTS. Some recent studies^26^ have shown that reparameterizations such as non-centered transformation can help address these issues but they are limited to hierarchical models with dependency between layers given by generalized linear model. In our efforts to sampling the posterior using NUTS, we found generating 500 samples from the posterior of the synthetic data, with 500 warmup iterations, takes 25 days finishing ~40–45 iterations per day on a Linux workstation with 3.0 GHz quad core processor. Supplementary Fig. 6 shows the results obtained from the NUTS samples and some diagnostics on these samples, which conveyed that the sampler has not been able to sample the whole posterior.

Even with the limitation of the MAP estimate potentially being a local optima, we demonstrated that the proposed approach offers a valuable application in validating clinical EZ hypothesis. When the MAP optimizer is initialized at the mean of priors, there are two possible outcomes: (i) if the prior and the likelihood support each other, the local optima near the mean of priors corresponds to the global optima, as evidenced by the good match between inferred EZ and clinical EZ hypothesis in Engel score I patients, and (ii) if the prior and likelihood are in disagreement, then the optimizer could converge to a local optima, which may or may not be the best solution. Even if it is only a local optima, the inferred EZ deviates from the clinical EZ hypothesis, as evidenced by the results on patients with Engel score III and IV, and synthetic data with bad hypothesis, providing valuable insight to the clinician so that the patient can be re-evaluated to improve the EZ hypothesis before any surgical resection. In the context of modeling neuroimaging data, different mathematical models or model configurations can be fitted to the observed data and then compared by information criteria such as Akaike information criterion (AIC) and Bayesian information criterion, to determine the best balance between model complexity and accuracy^27^. However, these classical information criteria are based on point estimates and are defined independently of prior knowledge. Alternatively, the fully Bayesian criteria such as Watanabe-AIC^28^ and leave-one-out cross-validation^29^, which are based on the whole posterior distribution rather than a point estimate, can be used to compare different EZ hypotheses. Measuring the out-of-sample prediction accuracy of our model with fully Bayesian evaluation of potential hypotheses regarding the degree of epileptogenicity across different brain regions remains to be investigated in a future study.

In clinical practice, EZ identification is computationally aided by signal analysis metrics such as epileptogenicity index (EI)^18^ or epileptogenicity maps^30^. The success and accuracy of these methods depends on optimal placement of electrodes^31^, as they do not take into account the problem of source mixing at sensors, which can lead to false positives when the implantation closely misses the EZ. In this study, EZ is identified based on the inferred source activity from the whole-brain network rather than just relying on the implanted regions. This is achieved by using a linear transformation to project the activity in source space into sensor space. Although such a projection addresses the issues of source mixing, it also introduces the problem of degeneracy for the inverse problem. Such degeneracy issues could be addressed if we have any prior knowledge about inherent structure of source dynamics to constrain the solution space. In the case of epilepsy, it is reasonable to assume that such a structure exists in the power profile of source activity. Specifically, the regions that seize would show a transient in power during a seizure. To exploit this structure, we have incorporated 2D Epileptor dynamics^3^ in the generative model as priors on source state transitions.

Combining a priori knowledge of the seizure dynamics, anatomical connections, and clinical expertise with observed intracranial recordings in a Bayesian framework, we have demonstrated that whole-brain network models could be inverted to build individualized in silico models of a patient’s seizure dynamics. Such a strategy to construct a personalized whole-brain model allows to refine clinical hypotheses and exploration of novel therapeutic techniques to improve epilepsy surgery outcome. Future extensions of this work could investigate reparameterizations of the generative model to sample the posterior efficiently using MCMC techniques.

## Methods

### Patient data

Model predictions are tested against a retrospective patient cohort containing 25 patients with drug-resistant epilepsy. SEEG and diffusion MRI data were collected from all 25 patients before surgery. Non-invasive T1-weighted images (magnetization prepared rapid acquisition gradient echo sequence, either with repetition time = 1.9 s and echo time = 2.19 ms or repetition time = 2.3 s and echo time = 2.98 ms, voxel size 1.0 × 1.0 × 1.0 mm) and diffusion MRI images (diffusion tensor imaging-MR sequence, either with angular gradient set of 64 directions, repetition time = 10.7 s, echo time = 95 ms, voxel size 1.95 × 1.95 × 2.0 mm, *b*-weighting of 1000 s mm^−2^, or with angular gradient set of 200 directions, repetition time = 3 s, echo time = 88 ms, voxel size 2.0 × 2.0 × 2.0 mm, *b*-weighting of 1800 s mm^−2^) are acquired using a Siemens Magnetom Verio 3T MR-scanner. SEEG data are collected using a 128-channel Deltamed system with a sampling rate of at least 256 Hz. SEEG recording are band-pass filtered between 0.16 and 97 Hz by a hardware filter. Patient details such as age, gender, preliminary clinical diagnosis, and Engel scores (clinical classification for epilepsy surgery) are given in Supplementary Table 1. The patients signed an informed consent form according to the rules of local ethics committee (Comiteé de Protection des Personnes Sud-Meéditerranée I). Accuracy of inferred EZ is evaluated by comparing it with the clinical EZ hypothesis that was used for the surgery. Clinical EZ hypothesis is generated by the clinicians by aggregating the information from semiology, non-invasive MRI, EEG, visual inspection of SEEG (invasive), and a data-driven signal-processing method metric called EI^18^.

#### Structural connectome

From the diffusion MRI data, a SC is built using the same reconstruction pipeline as in Hashemi et al.^22^ but using a different parcellation with a finer spatial resolution. Briefly, the pipeline involves the following: (a) parcellation of brain anatomy from T1-weighted images using FreeSurfer v6.0.0^32^; (b) coregistration with diffusion-weighted images using flirt^33^ from FSL package in version 6.0; (c) estimating fiber orientation distributions using dwi2fod tool^34,35^; (d) generate fiber tracts using iFOD2 probabilistic tractography algorithm^36^; and (e) building the connectome using tck2connectome tool. Tractography is performed using MRtrix package in version 0.3.15. In this study, we used the Destrieux parcellation^37^ containing 164 brain regions with 74 cortical regions per hemisphere and 16 subcortical regions. Region abbreviations, labels, and indices are provided in Supplementary Table 2. The connectome is normalized such that the maximum value is equal to one.

#### Source-to-sensor space transformation

The implanted intracranial electrodes record the local field potential generated by the neuronal tissue in its neighborhood. We assume a linear relation between the source activities and the generated signals,

1 $${s}_{i}(t)=\mathop{\sum }\limits_{j=1}^{N}{G}_{ij}{\phi }_{j}(t),$$

where *s*_*i*_(*t*) is the signal at sensor *i*, *ϕ*_*j*_(*t*) is the source activity in region *j*, and *G*_*ij*_ is the coefficient of transformation. To calculate it, we represent the cortical regions by their triangulated pial surfaces and the subcortical regions by their triangulated enclosing surfaces as obtained from the reconstruction by FreeSurfer^32^. Assuming that the generated signal decays with square of the distance from the source, the coefficient is

2 $${G}_{ij}=\mathop{\sum}\limits_{k\in {V}_{j}}\frac{c\,{A}_{k}}{| {\overrightarrow{x}}_{i}^{s}-{\overrightarrow{x}}_{k}^{v}{| }^{2}},$$

where *V*_*j*_ is the set of all vertices on the triangulate surface of region *j*, *c* is the scaling coefficient, *A*_*k*_ is the surface associated with vertex *k*, ${\overrightarrow{x}}_{i}^{s}$ is the position of the sensor *i*, and ${\overrightarrow{x}}_{k}^{v}$ is the position of the vertex *k*. We have not taken into account the dependency of the source-to-sensor decay on the orientation of the neuronal tissue. Although the orientation plays an important role for the local field potential generated by the cortical tissue where a clear geometrical arrangement of the neurons exist^38,39^, it is difficult to quantify this effect for the subcortical structures with their diverse structural arrangements. Thus, due to the lack of information about the orientation in subcortical structures, we have chosen to omit the orientation dependency.

### Synthetic data

**Fig. 9:**
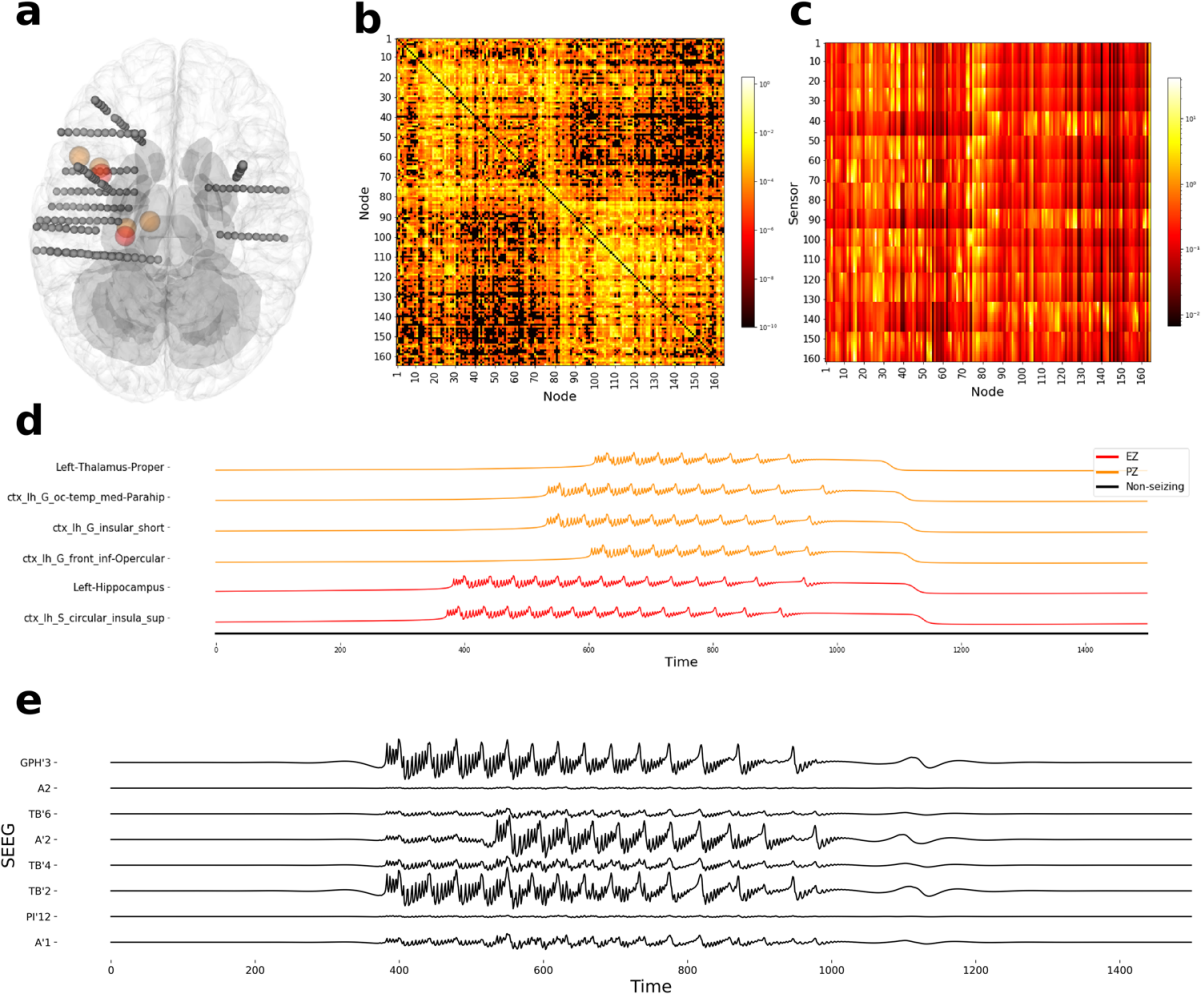
Simulated seizure data generated using structural connectivity and electrode implantation of patient BT from the retrospective cohort. **a** Top view of electrode implantation. Spheres colored in red and orange represent the centers of regions in the EZ and PZ, respectively. **b** Structural connectivity in log scale. **c** Gain matrix, transformation matrix from source-to-sensor space, in log scale. **d** Simulated local field potential (*x*_1_(*t*) + *x*_2_(*t*)) depicting the seizure propagation pattern. **e** Simulated SEEG activity from eight channels. EZ, epileptogenic zone; PZ, propagation zone; SEEG, stereotactic electroencephalography.

To compare model-predicted seizure propagation with the ground truth at the source space, a synthetic data set is generated using coupled five-dimensional (5D) Epileptor model (Eq. (3)). Epileptor is a phenomenological model of seizure dynamics and is shown to realistically reproduce key features of epileptic seizure dynamics such as onset, progression, and offset in different species^2^. Mathematically, the Epileptor model is defined by five state variables coupling two oscillatory dynamical systems on three different timescales: on the fastest timescale, variables *x*_1_ and *y*_1_ account for fast discharges during the ictal state. On the intermediate timescale, variables *x*_2_ and *y*_2_ represent the slow spike-and-wave oscillations. On the slowest timescale, the variable *z*, described as permittivity variable, as it represents the ability of the model to resist seizure-triggering events and controls the transition between interictal and ictal states. Proix et al.^3^ demonstrated that simple and complex seizure recruitment among brain regions can be modeled by coupling Epileptor nodes with a permittivity-based coupling. Following the same approach, we used structural connectivity (Fig. 9b) from a randomly selected retrospective patient (BT) to couple 5D Epileptor nodes (given by Eq. (3) with *N* = 164) to generate a synthetic seizure propagation pattern. The dynamics of node *i* are thus described by the following coupled differential equations.

3 $${\dot{x}}_{1,i}=	\, {y}_{1,i}-{f}_{1}({x}_{1,i},{x}_{2,i})-z+{I}_{1}\\ {\dot{y}}_{1,i}=	\, 1-5{x}_{1,i}^{2}-{y}_{1,i}\\ \dot{{z}_{i}}= 	\, \frac{1}{{\tau }_{0}}(4({x}_{1,i}-{x}_{0})-{z}_{i}-\mathop{\sum }\limits_{j=1}^{N}K{C}_{ij}({x}_{1,j}-{x}_{1,i}))\\ {\dot{x}}_{2,i}= 	 -\!{y}_{2,i}+{x}_{2,i}-{x}_{2,i}^{3}+{I}_{2}+0.002g({x}_{1,i})-0.3({z}_{i}-3.5)\\ {\dot{y}}_{2,i}= 	\, \frac{1}{{\tau }_{2}}(-{y}_{2,i}+{f}_{2}({x}_{2,i}))$$

where

$${f}_{1}({x}_{1,i},{x}_{2,i})=	\, \left\{\begin{array}{ll}{x}_{1,i}^{3}-3{x}_{1,i}^{2}\hfill&\,{{\mbox{if}}}\,{x}_{1,i}\, < \, 0\hfill\\ ({x}_{2,i}-0.6{\left({z}_{i}-4\right)}^{2}){x}_{1,i}\quad &\,{{\mbox{if}}}\,\,{x}_{1,i}\,\ge\, 0\end{array}\right.\\ {f}_{2}({x}_{2,i})=	\, \left\{\begin{array}{ll}0\hfill &\,{{\mbox{if}}}\,{x}_{2,i}\, < -\!0.25\hfill\\ 6({x}_{2,i}+0.25)\quad &\,{{\mbox{if}}}\,{x}_{2,i}\ge -\!0.25\end{array}\right.\\ g({x}_{1,i})=	\, \int_{-{t}_{0}}^{t}{\exp }^{-\gamma (t-\tau )}{x}_{1,i}(\tau )dt$$

where *τ*_0_ = 2857, *τ*_2_ = 10, *I*_1_ = 3.1, *I*_2_ = 0.45, and *γ* = 0.01. The parameter *x*_0_ represents excitability of a brain region and an isolated Epileptor node produces seizure if *x*_0_ > −2.1. Further details regarding linear stability analysis and biological interpretation of parameters are provided in refs. ^2,3,5^.

For the synthetic seizure used in this study, two brain regions (left hippocampus and ctx-lh-S-circular-insula-sup), within the vicinity of electrode implantation, are chosen as the EZ with *x*_0_ = −1.8. Four regions (ctx-lh-G-front-inf-Opercular, ctx-lh-G-insular-short, ctx-lh-G-oc-temp-med-Parahip, and left thalamus proper), which are anatomically strongly connected to regions in EZ, are chosen as part of the PZ with *x*_0_ = −2.3. In all other regions, *x*_0_ is set to −3.0. Simulated SEEG data (Fig. 9e) is then generated by projecting the local field potential given by *x*_1_(*t*) + *x*_2_(*t*) (Fig. 9d) into sensor space using a linear transformation (Fig. 9c). Simulations are performed in TVB^14^ using Heun integration scheme with a time step size of 0.04 ms for 2500 ms.

### Data preprocessing/feature extraction

A spatio-temporal seizure propagation can be characterized in terms of seizure-onset time and seizure length of all the regions recruited by the seizure. The log power profile of all brain regions captures both these features and it can be modeled using the reduced 2D Epileptor^3^. Inference over reduced 2D Epileptor allows for faster inversion, compared to inversion of 5D Epileptor, while enabling us to predict the envelope of fast discharges during the ictal states^22^. Hence, raw SEEG data are preprocessed to extract SEEG log power. Preprocessing involves high-pass filtering raw SEEG data from 10 Hz, computing the power over a sliding window, applying a log transformation, and finally a low-pass filter, tuned per each patient, is applied for smoothing out any short spikes in the data. In order to reduce the computational cost of fitting, the SEEG log power time series is down-sampled to 300 time points. Data augmentation is a common technique in machine learning, to improve optimization when the observations are sparse. Hence, apart from SEEG log power, total power in each sensor (i.e., the second sample moment of each sensors log power time series) is used as an augmented data feature.

### Generative model

In a Bayesian paradigm, the generative model is a statistical model over the observed and latent variables. Here, it is defined by the joint probability density over the combined space of 2D Epileptor parameters, hidden states, and the observations. Using the chain rule of probability, this joint probability density can be factorized as the product of likelihood and priors:

4 $$P(\theta ,{{{{{{{\bf{Y}}}}}}}},{{{{{{{\bf{D}}}}}}}})=P({{{{{{{\bf{D}}}}}}}}| \theta ,{{{{{{{\bf{Y}}}}}}}})P(\theta ,{{{{{{{\bf{Y}}}}}}}})$$

where

$${{{\bf{D}}}}=	\, ({{{{{\bf{S}}}}}},\overrightarrow{\rho })\\ {{{\bf{S}}}}=	\, \left(\begin{array}{llll}{s}_{1}({t}_{1})&{s}_{2}({t}_{1})&\cdots &{s}_{M}({t}_{1})\\ {s}_{1}({t}_{2})&{s}_{2}({t}_{2})&\cdots &{s}_{M}({t}_{2})\\ \vdots &\vdots &\ddots &\vdots \\ {s}_{1}({t}_{T})&{s}_{2}({t}_{T})&\cdots &{s}_{M}({t}_{T}) \end{array}\right)_{T\times M}\\ {{{\bf{Y}}}}=	\, \left(\begin{array}{llll}{x}_{1}({t}_{1}) & {x}_{1}({t}_{2}) & \cdots & {x}_{1}({t}_{T})\\ {z}_{1}({t}_{1}) & {z}_{1}({t}_{2}) & \cdots & {z}_{1}({t}_{T})\\ \vdots & \vdots & \ddots & \vdots \\ {x}_{N}({t}_{1}) & {x}_{N}({t}_{2}) & \cdots & {x}_{N}({t}_{T})\\ {z}_{N}({t}_{1}) & {z}_{N}({t}_{2}) & \cdots & {z}_{N}({t}_{T}) \end{array}\right)_{T\times M}\\ \theta =	\, \left({\overrightarrow{x}}_{0},\overrightarrow{x}({t}_{0}),\overrightarrow{z}({t}_{0}),K,{\tau }_{0},\alpha ,\beta ,{\epsilon }_{1},{\epsilon }_{2}\right)$$

**S** is the matrix of random variables with element *s*_*i*_(*t*_*j*_) representing SEEG log power from sensor *i* at time *t*_*j*_. $\overrightarrow{\rho }$ represents the augmented data feature, total power in each sensor. **Y** is a matrix of random variables representing the evolution of unobserved source states. In this study, the source state is given by the 2D Epileptor variables *x* and *z* (Eq. (6)). In the 2D Epileptor model, *x* can be interpreted as a proxy variable to the source log power and *z* is a slow permittivity variable, which determines how close the system is to seizure threshold^2^. Thus, column *j* of the matrix **Y** represents the unobserved source state at time *t*_*j*_, where element *x*_*i*_(*t*_*j*_) is the log power of region *i* at time *t*_*j*_ and element *z*_*i*_(*t*_*j*_) is the slow permittivity variable of region *i* at time *t*_*j*_. *θ* is a vector of random variables, of length 498, representing all the free parameters that are inferred. The vector ${\overrightarrow{x}}_{0}$, of length 164, represents the excitability parameter of all regions. $\overrightarrow{x}({t}_{0}),\overrightarrow{z}({t}_{0})$, with 164 elements in each, are the initial source states of all brain regions. *K* and *τ*_0_ are scalar parameters in the Epileptor model representing global coupling and timescale (see Eq. (6)). *α* and *β* are auxiliary scalar parameters representing the scaling and offset of model-predicted SEEG log power, respectively. *ϵ*_1_ and *ϵ*_2_ represent the observation noise strength in SEEG log power and the augmented data feature, respectively. *T* is the number of samples in time, *N* is the number of regions in the parcellation, and *M* is the number of sensors. By Bayes’ theorem, the joint probability density (Eq. (4)) is proportional, up to a normalizing constant, to the posterior probability density of Epileptor parameters and hidden states conditioned on observed data:

5 $$P(\theta ,{{{{{{{\bf{Y}}}}}}}}| {{{{{{{\bf{D}}}}}}}})\propto P({{{{{{{\bf{D}}}}}}}}| \theta ,{{{{{{{\bf{Y}}}}}}}})P(\theta ,{{{{{{{\bf{Y}}}}}}}})$$

#### Priors

One of the advantages of Bayesian inference is that any prior knowledge such as parameter constraints can be incorporated easily into the model. Moreover, priors can be adjusted to be strong or weak based on the confidence in that knowledge. In this study, dynamics of source log power are governed by coupled 2D Epileptor equations given below.

6 $$\begin{array}{rcl}{\dot{y}}_{i}&=&\left[\begin{array}{c}{\dot{x}}_{i}\\ {\dot{z}}_{i}\\ \end{array}\right]=\left[\begin{array}{c}1-{x}_{i}^{3}-2{x}_{i}^{2}-{z}_{i}+{I}_{1}\\ \frac{1}{{\tau }_{0}}\left(4({x}_{i}-{x}_{0})-{z}_{i}-\mathop{\sum }\nolimits_{j = 1}^{N}K{C}_{ij}({x}_{j}-{x}_{i})\right)\end{array}\right]\end{array}$$

2D Epileptor dynamics are embedded into the prior as the transition probabilities on hidden source states as:

$$P(\theta ,{{{{{\bf{Y}}}}}})=	\, P({{{{{\bf{Y}}}}}}| \theta )P(\theta )\\ P({{{{{\bf{Y}}}}}}| \theta )=	\, P(\overrightarrow{y}({t}_{0})| \theta )\mathop{\prod }\limits_{j=1}^{T}P(\overrightarrow{y}({t}_{j})| \overrightarrow{y}({t}_{j-1}),\theta )$$

where, $P(\overrightarrow{y}({t}_{j})| \overrightarrow{y}({t}_{j-1}),\theta )$ represents the state transition probability from time *t*_*j*−1_ to *t*_*j*_ given the current state $\overrightarrow{y}({t}_{j-1})$ and Epileptor parameters *θ*.

In a more general framework, for systems described by nonlinear stochastic differential equations (SDE) of the generic form *dy* = *f*(*y*, *t*)*dt* + *L*(*y*, *t*)*dβ*, where *f* is the drift function, *L* is the dispersion matrix, and *β* is Brownian motion with diffusion matrix *Q*, the state transition probability density is usually intractable. In such cases, the transition density can be approximated using SDE simulation and discretization methods^40^, which is valid for sufficient regularity and small step size. Under these conditions, a common choice for the approximate transition density is a normal distribution with mean given by numerically integrating the dynamical model from *t*_*j*−1_ to *t*_*j*_, i.e.,

7 $$P(\overrightarrow{y}({t}_{j})| \overrightarrow{y}({t}_{j-1}),\theta )={{{{{{{\mathcal{N}}}}}}}}(f(\overrightarrow{y}({t}_{j-1}),\theta ),\epsilon )$$

where the function $f(\overrightarrow{y}({t}_{j-1}),\theta )$ represents the state on the 2D Epileptor trajectory after a small time step departing from $\overrightarrow{y}({t}_{j-1})$ and *ϵ* represents the SD of the normal distribution (or equivalently noise in state dynamics). For small noise, state space exploration would be limited to local variations around the deterministic trajectory, consistent with the above choice of normal distribution. If the stochastic properties of the system (such as *L* and *Q*) were known a priori, then *ϵ* could be precomputed based on the discretization method, else it would have to be inferred as one of the parameters of the inference procedure.

In our case, the state transition is a deterministic transformation of the current state and model parameters^41–43^, which significantly reduces the number of parameters that need to be inferred, as the latent states are deterministically computed given the model parameters. Mathematically, this is equivalent with the limit *ϵ* → 0 in Eq. (7) leading to

8 $$P(\overrightarrow{y}({t}_{j})| \overrightarrow{y}({t}_{j-1}),\theta )=\delta (\overrightarrow{y}({t}_{j})-f(\overrightarrow{y}({t}_{j-1}),\theta ))$$

As the state dynamics are described by an ordinary differential equation (ODE), $f(\overrightarrow{y}({t}_{j-1}),\theta )$ can be solved by any ODE solver. We used a fourth-order Runge–Kutta method with a time step of 0.1.

Based on the dependency structure (Supplementary Fig. 7), prior probability density over Epileptor parameters and the auxiliary parameters can be factorized as:

$$P(\theta )=	\, P({\overrightarrow{x}}_{0},\overrightarrow{x}({t}_{0}),\overrightarrow{z}({t}_{0}),K,{\tau }_{0},\alpha ,\beta ,{\epsilon }_{1},{\epsilon }_{2})\\ =	\, P(K)P({\tau }_{0})P(\alpha )P(\beta )P({\epsilon }_{1})P({\epsilon }_{2})\mathop{\prod }\limits_{i=1}^{N}P({x}_{0,i})P({x}_{i}({t}_{0}))P({z}_{i}({t}_{0}))$$

All the priors are defined to be either normal or truncated normal distributions. The mean of the priors is set based on the a priori knowledge about the dynamical system properties of the Epileptor model and the clinical hypothesis of the EZ. The timescale parameter (*τ*_0_) is set by analyzing simulations, so that the transition to seizure state is smooth and not a sudden jump. This choice is made, as the fitted data features are smoothed in preprocessing. Prior values of initial conditions ($\overrightarrow{x}({t}_{0}),\overrightarrow{z}({t}_{0})$) are set close to a stable fixed point of a single Epileptor node. As we have no information about the global coupling parameter (*K*), its mean is set to 1, i.e., no scaling is assumed on the connectivity between regions. For the auxiliary parameters, as no information is available, their mean is set such that no assumptions are made regarding the amplitude scaling (*α*) and offset (*β*) between model-predicted and -simulated SEEG log power. Observation noise parameters (*ϵ*_1_, *ϵ*_2_) are set based on a comparison between a few simulated and observed SEEG log power. Mean and SD values of all these parameters are given in Table 1. Epileptor parameter *x*_0,*i*_ represents excitability of tissue in brain region *i*. An isolated Epileptor node would trigger seizures if *x*_0_ > −2.1. Thus, clinical hypothesis about the EZ is incorporated as a weakly informative prior on the excitability parameter as given by:

$$P({x}_{0,i})=\left\{\begin{array}{ll}{{{{{{{\mathcal{N}}}}}}}}(-1.5,1)\quad &\,{{\mbox{if region}}}\,i\in \,{{\mbox{EZ Hypothesis}}}\,\\ {{{{{{{\mathcal{N}}}}}}}}(-3.0,1)\quad &\,{{\mbox{otherwise}}}\,\hfill\end{array}\right.$$

where, ${{{{{{{\mathcal{N}}}}}}}}$ represents a normal distribution.

**Table 1:** Mean and SD of the normal prior probability densities.

Parameter	Mean	SD
*K*	1	10
*τ*_0_	20	10
*x*_*i*_(*t*_0_)	− 2.0	10
*z*_*i*_(*t*_0_)	3.5	10
*α*	1	10
*β*	0	10
*ϵ*_1_	1	10
*ϵ*_2_	1	10

Mean and SD of the normal prior probability densities of global coupling (*K*), time scaling parameter (*τ*_0_), initial conditions (*x*_*i*_(*t*_0_), *z*_*i*_(*t*_0_)), amplitude scaling (*α*) and offset (*β*) of SEEG log power, observation noise strength in SEEG log power (*ϵ*_1_), and total sensor power (*ϵ*_2_). For parameters that are defined only in the probability, densities are truncated below at zero.

#### Likelihood

Likelihood describes the probability of observed data under a particular realization of the parameters. It is defined as:

9 $$P({{{{{{{\bf{S}}}}}}}}=\hat{{{{{{{{\bf{S}}}}}}}}},\overrightarrow{\rho }=\hat{\rho }| {{{{{{{\bf{Y}}}}}}}},\theta )=P({{{{{{{\bf{S}}}}}}}}=\hat{{{{{{{{\bf{S}}}}}}}}}| {{{{{{{\bf{Y}}}}}}}},\theta )P(\overrightarrow{\rho }=\hat{\rho }| {{{{{{{\bf{S}}}}}}}}=\hat{{{{{{{{\bf{S}}}}}}}}},{{{{{{{\bf{Y}}}}}}}},\theta )$$

where, $\hat{{{{{{{{\bf{S}}}}}}}}},\hat{\rho }$ represent a particular realization of ${{{{{{{\bf{S}}}}}}}},\overrightarrow{\rho }$, i.e., the SEEG log power and total sensor power computed from the SEEG recordings of a patient. To simplify notation, the realizations are not shown further. *P*(**S**∣**Y**, *θ*) represents the probability of observed SEEG log power given that the latent source states are **Y**. This is defined as a normal distribution with mean given by projecting the source state to sensor space:

$$P({{{{{\bf{S}}}}}}| {{{{{\bf{Y}}}}}},\theta )=	\, \mathop{\prod }\limits_{i=1}^{M}\mathop{\prod }\limits_{j=1}^{T}P({s}_{i}({t}_{j})| \overrightarrow{x}({t}_{j}),\theta )\\ P({s}_{i}(t)| \overrightarrow{x}(t),\theta ) \sim 	\, {{{{{\mathcal{N}}}}}}(\alpha {{{{\mathrm{log}}}}}\,\langle {G}_{i}\ ,{e}^{\overrightarrow{{x}_{t}}}\rangle +\beta ,{\epsilon }_{1})$$

where, 〈. , . 〉 represents an inner product. *G*, known as the gain matrix, is the linear transformation from source-to-sensor space. *α* and *β* are auxiliary parameters, which account for the scaling and offset, respectively, in the observations. $P(\overrightarrow{\rho }| {{{{{{{\bf{S}}}}}}}})$ represents the probability of the augmented data feature, total sensor power, given the observed SEEG log power and the latent states. It is defined as:

$$P(\overrightarrow{\rho }| {{{{{\bf{S}}}}}},{{{{{\bf{Y}}}}}},\theta )=	\, \mathop{\prod }\limits_{i=1}^{M}P({\rho }_{i}| {{{{{\bf{S}}}}}},\theta )\\ P({\rho }_{i}| {{{{{\bf{S}}}}}},\theta ) \sim 	\, {{{{{\mathcal{N}}}}}}\left(\frac{1}{T}\mathop{\sum }\limits_{j=1}^{T}{\left({s}_{i}({t}_{j})\right)}^{2},{\epsilon }_{2}\right)$$

### Model inversion

By construction, maxima of the posterior density (Eq. (5)) corresponds to the set of parameters that best explain the observed data. Maxima of the posterior density are identified using a quasi-Newton optimization algorithm L-BFGS^16^. Optimization is initialized at the mean of the priors and is run till convergence. Convergence is monitored according to the following three criteria: (a) density convergence: change in unnormalized log posterior density is <10^−12^; (b) gradient convergence: Euclidean norm of gradient is <10^−8^; and (c) parameter convergence: change in parameter value is <10^−8^. Numerical implementation of the generative model and model inversion are performed using a PPL Stan^13^. On a Linux workstation with 3.0 GHz quad core processor model inversion, using MAP took, on average, 1 h for optimization to converge.

### Identifying EZ and PZ

EZ is the network of brain regions where seizure originates and PZ is the network of brain regions that are later recruited by the seizure due to the coupling with regions in EZ. Although in an isolated Epileptor node seizure would originate when the excitability parameter (*x*_0_) is greater than te bifurcation threshold value −2.1, such a threshold does not exist in a network of coupled Epileptors. Hence, in this study we used seizure-onset times of the regions to identify EZ and PZ. First, onset times are estimated by finding the time instant where depolarization shift occurred in the model-predicted activity of the fast variable (*x*). Next, all regions with onset times within a tolerance, *t*_*ϵ*_, of the earliest onset time, *t*_*λ*_, are classified as EZ. All other regions with onset times beyond *t*_*λ*_ + *t*_*ϵ*_ are classified as PZ.

### Reporting summary

Further information on research design is available in the Nature Research Reporting Summary linked to this article.

## Supplementary information

Supplementary Information

Reporting Summary

## Data Availability

The patient data sets cannot be made publicly available due to the data protection concerns. The synthetic data used in this study are available in figshare with the identifier doi:10.6084/m9.figshare.16628332.v1^44^.

## Code availability

The Stan code used in this study is available in figshare with the identifier 10.6084/m9.figshare.16636411.v1^45^. Stan in version 2.23.0 is used for performing MAP.
