# Supplementary material for: Identifying spatio-temporal seizure propagation patterns in epilepsy using Bayesian inference
Source: Commun Biol. 2021 Nov 1;4:1244. doi: 10.1038/s42003-021-02751-5 (PMC8560929; doi:10.1038/s42003-021-02751-5)
Supplement: Supplementary file 1 — Supplementary Information [file 42003_2021_2751_MOESM1_ESM.pdf]

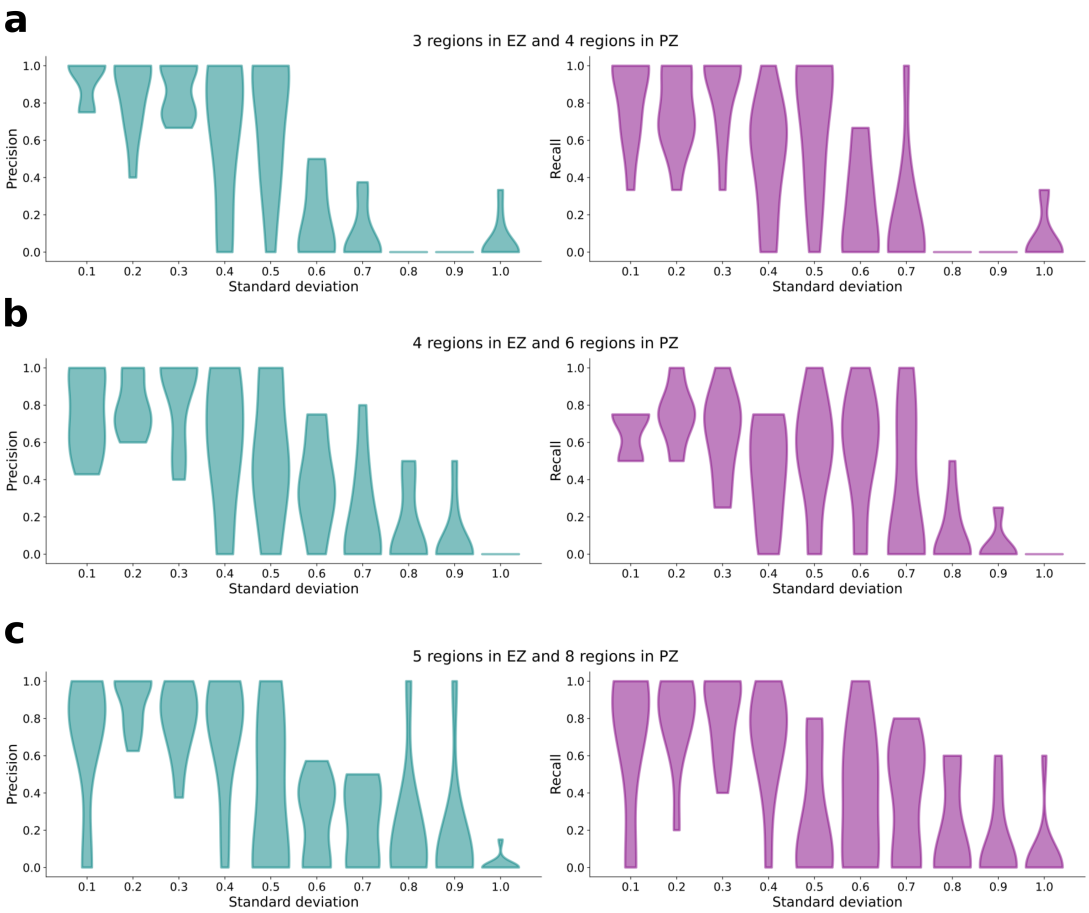

Supplementary Figure 1: Accuracy of estimated EZ in three different synthetic datasets across different initial conditions. (a) Precision and recall of estimated EZ, in fitting a synthetic dataset with 3 regions in EZ and 4 regions in PZ, as the standard deviation of the proposal distribution of initial conditions is increased from 0.1 to 1.0. (b) Same as panel a except the dataset contains 4 regions in EZ and 6 regions in PZ. (c) Same as panel a except the dataset contain 5 regions in EZ and 8 regions in PZ

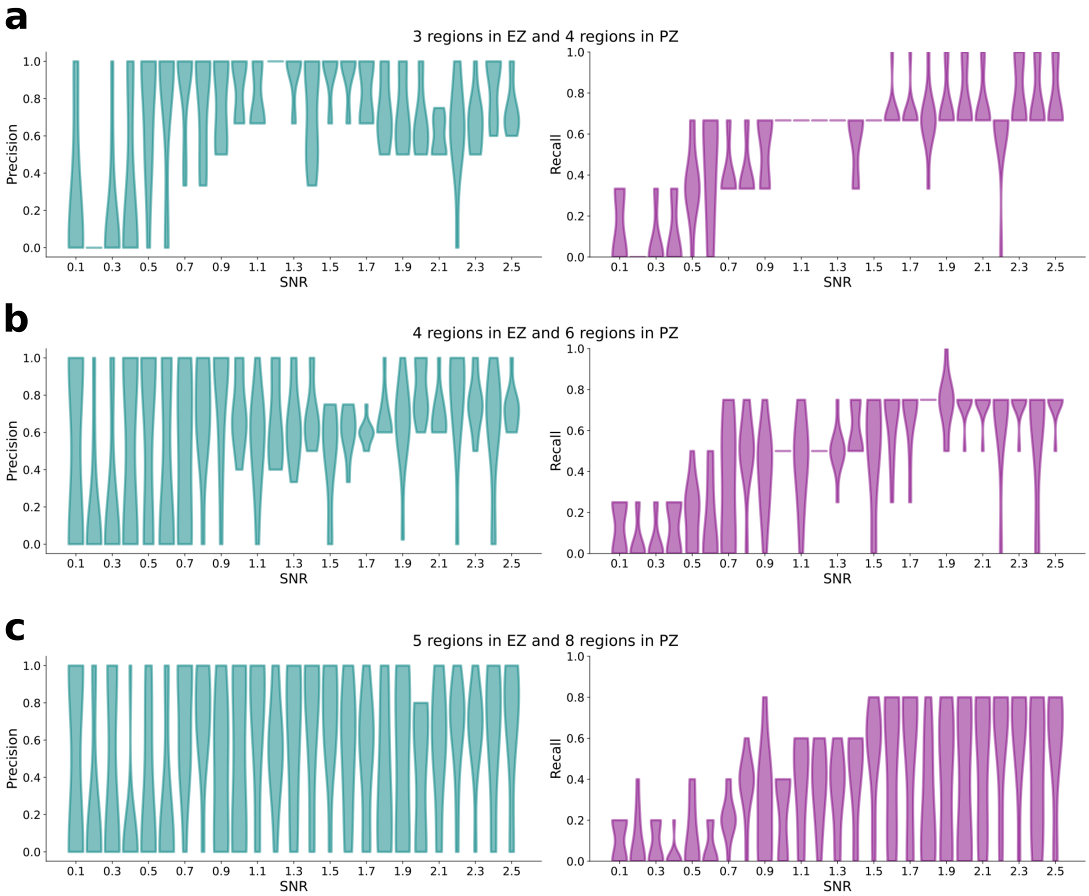

Supplementary Figure 2: Accuracy of estimated EZ in three different synthetic datasets across different observation noise. (a) Precision and recall of estimated EZ, in fitting a synthetic dataset with 3 regions in EZ and 4 regions in PZ, as the signal to noise ratio in the observations is increased from 0.1 to 2.5. (b) Same as panel a except the dataset contains 4 regions in EZ and 6 regions in PZ. (c) Same as panel a except the dataset contain 5 regions in EZ and 8 regions in PZ

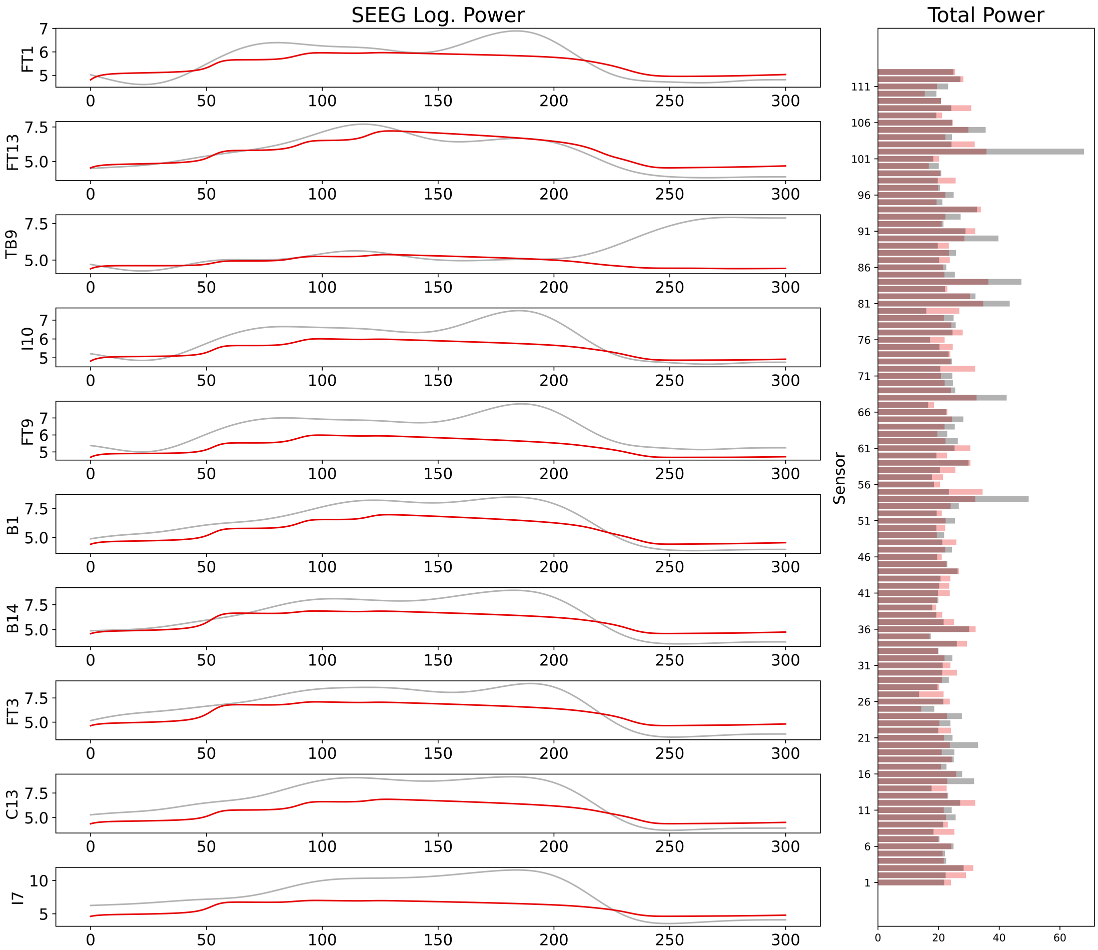

Supplementary Figure 3: Comparison of model predicted sensor power (left) of 10 sensors with highest power and the augmented data feature:total sensor power (right) for patient LMA with Engel score I. Observations are shown in gray and model predictions are shown in red

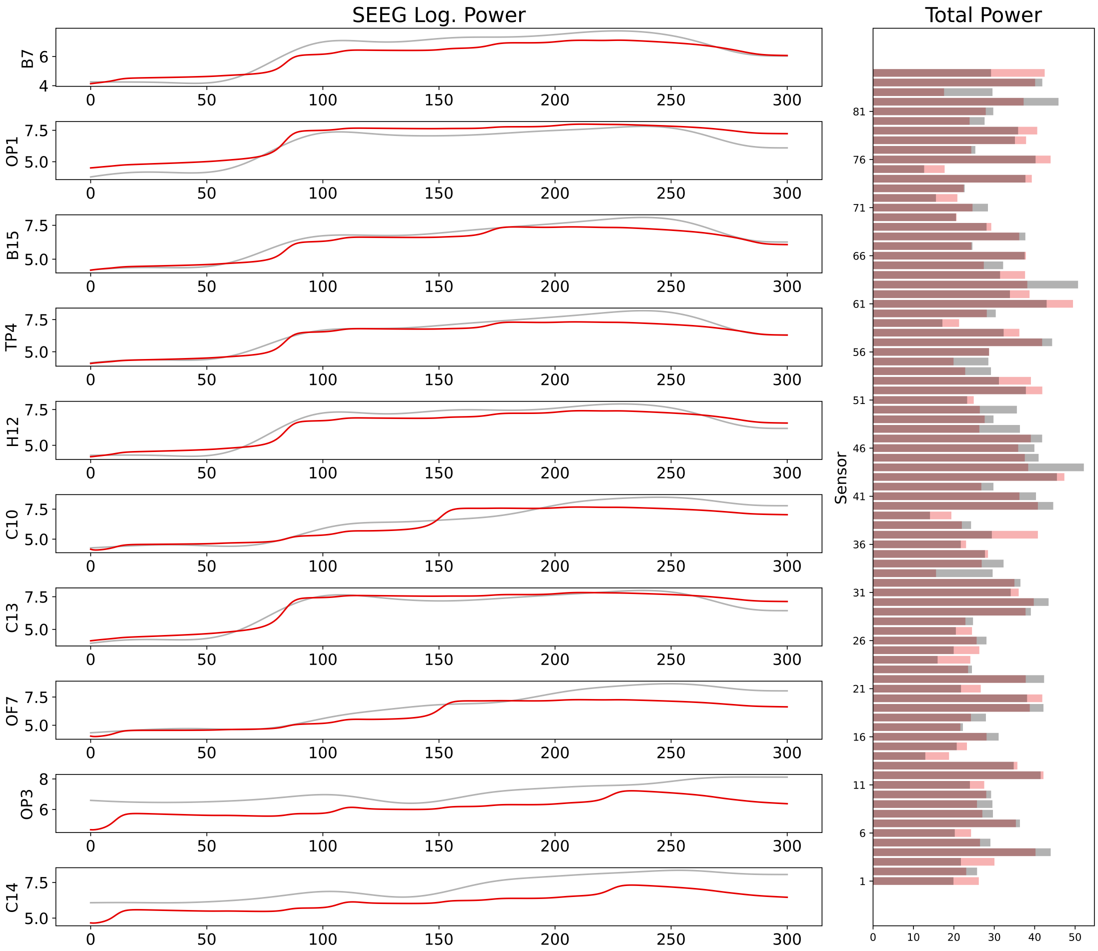

Supplementary Figure 4: Same as Fig. 3 except for patient FC with Engel score IV

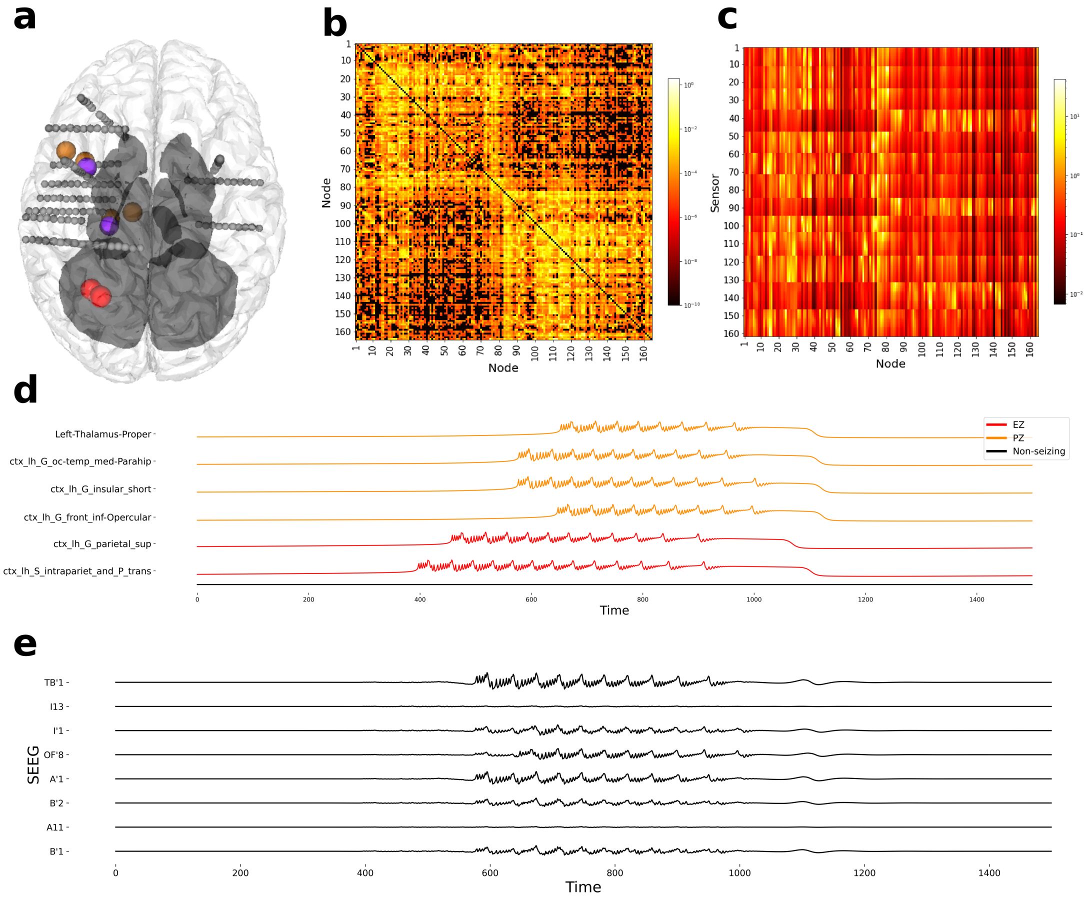

Supplementary Figure 5: Simulated seizure data generated using SC and electrode implantation of patient BT from the retrospective cohort. (a) Top view of electrode implantation. Spheres colored in red and orange represent the centers of regions in the EZ and PZ, respectively. Spheres in purple represent the regions in EZ hypothesis used in Fig. 8 in main text. (b) Structural connectivity in log scale. (c) Gain matrix, transformation matrix from source to sensor space, in log scale. (d) Simulated local field potential $(x_1(t) + x_2(t))$ depicting the seizure propagation pattern (e) Simulated SEEG activity from 8 channels.

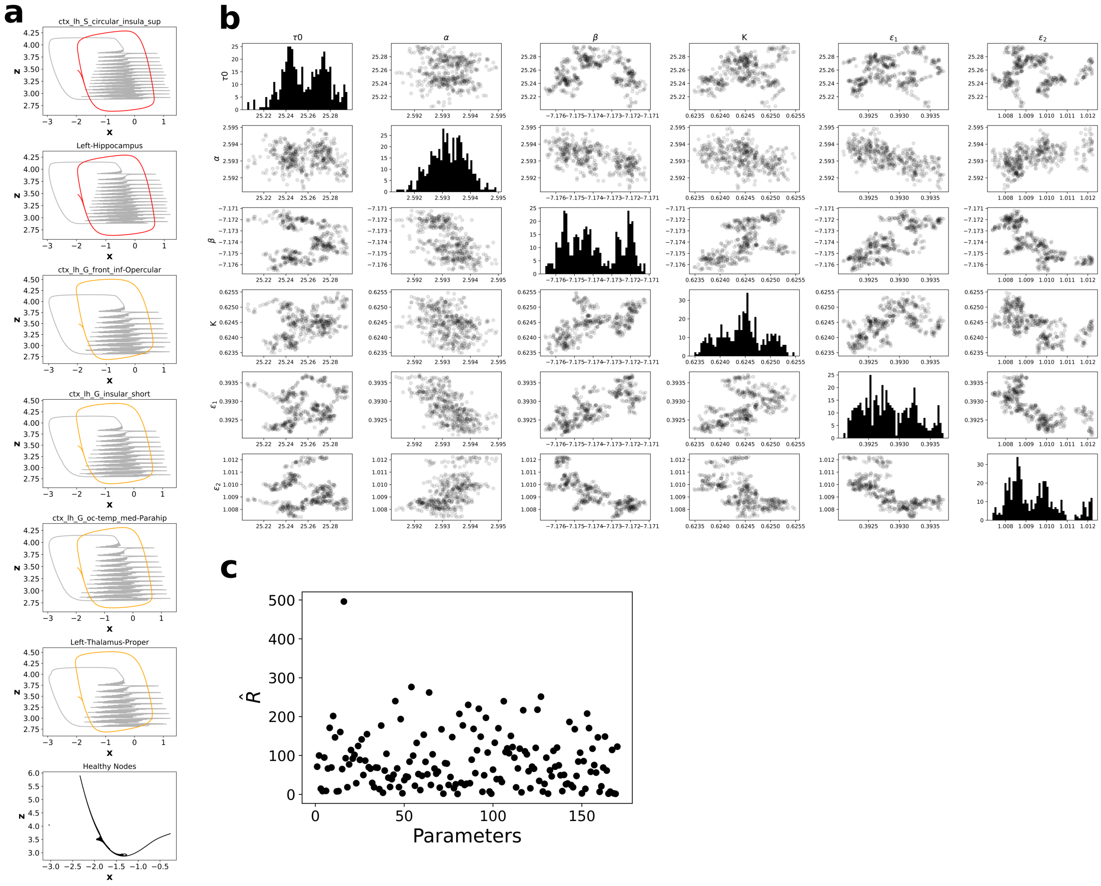

Supplementary Figure 6: Results of inference with NUTS sampler. (a) Same as Fig. 4A in main text except the inferred source activity represents the mean of 500 samples from posterior, (b) Pair plots of all inferred scalar parameters, (c) Scale reduction statistic, $\hat{R}$, of all the inferred parameters computed from 1000 samples of two MCMC chains. If the MCMC chains have converged then $\hat{R}$ will be close to one. The higher $\hat{R}$ values obtained here indicate that the chains have not yet converged.

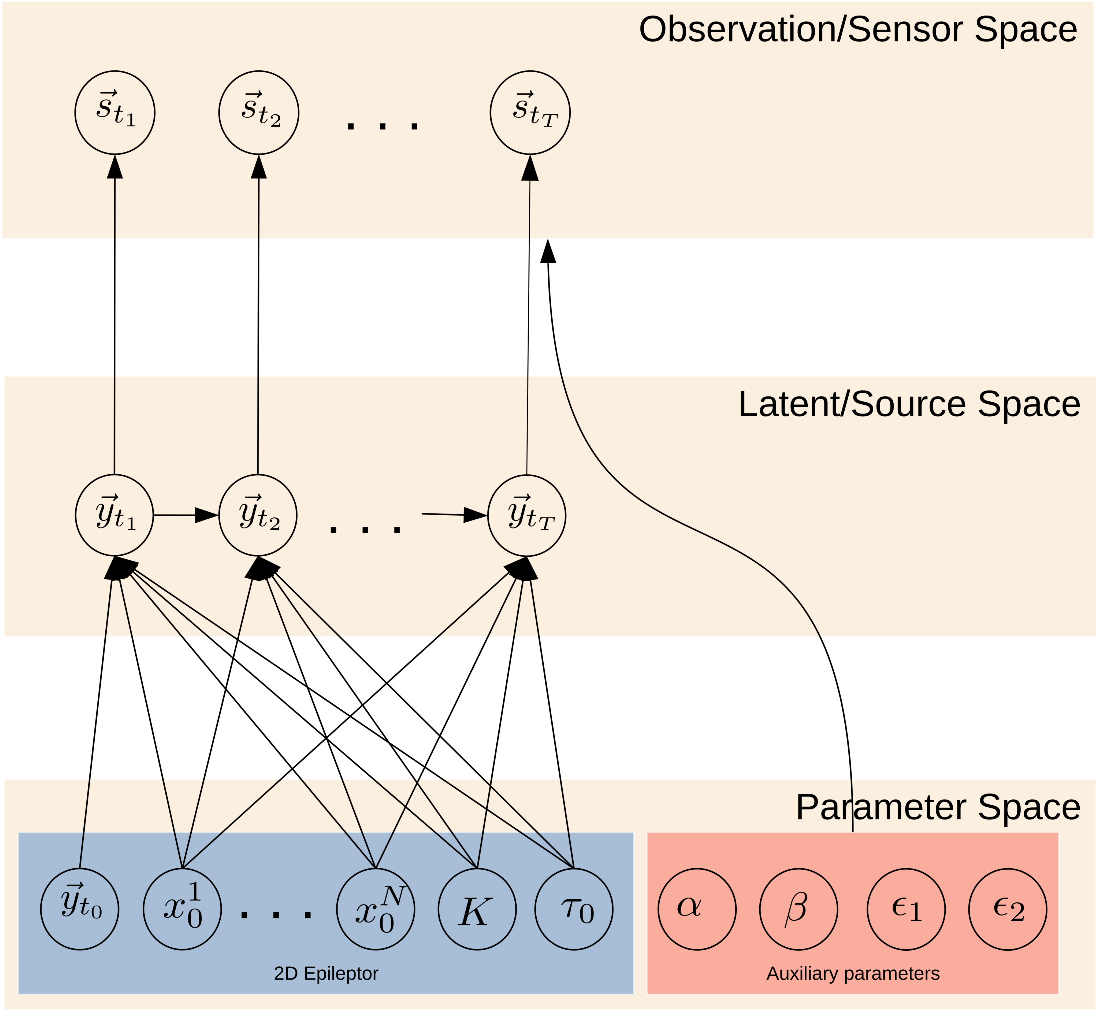

Supplementary Figure 7: Probabilistic graphical model depicting the dependency structure between various random variables of hierarchical model given in Eq. 4 in main text. The hierarchical model consists of three levels of interdependence. The dependency between random variables in various levels of the hierarchy is given by the directed arrows. Parameter space consisting of all 2D Epileptor model parameters and auxiliary parameters (θ) form the bottom level of the hierarchy. The middle level consists of the unobserved source states ($\vec{y}_{t_i}$) which are dependent on random variables in parameter space. At the top of the hierarchy are the observable data features ($\vec{s}_{t_i}$) of SEEG activity which conditioned on source states are independent of the levels below.

Supplementary Table 1: Retrospective patient details.
Abbreviations: AVM=arteriovenous malformation; FCD=focal cortical dyplasia; NA=not applicable; L=left; R=right.

Patient ID	Gender	Age at epilepsy onset (y)	Epilepsy duration (y)	Epilepsy type	MRI	Histopathology	Hemisphere	Engel score
BT	F	31	3	Temporo-insular	Normal	Hippocampal sclerosis	R	I
MG	M	23	13	Temporo-frontal	R temporo-occipital scar	FCD1a	R	I
BJ	F	23	3	Temporal	R temporal mesial ganglioglioma	Ganglioglioma	R	I
RD	M	55	5	Temporal	Normal	Slight gliosis	R >L	III
DMC	F	38	8	Temporal	L amygdala enlargement	Slight gliosis	L	III
BA	F	11	34	Bifocal: parietal mesial and temporo-basal	Unknown R parietal lesion	Rosenthal fibers; slight gliosis	R	III
CMN	F	27	18	Temporal	L hippocampal sclerosis	Hippocampal sclerosis	L	I
GR	F	27	14	Frontal	L frontal scar (abcess)	Gliosis	L	IV
LK	M	2	17	Frontal	Normal	Slight gliosis	L	I
VC	F	5	18	Premotor	Normal	FCD2b	L	II
MK	M	5	21	Temporal	L temporo-polar hypothrophy and hippocampal sclerosis	Hippocampal sclerosis; gliosis	L	I
LMA	F	17	10	Temporal	Normal	Hippocampal sclerosis	R	I
JC	F	9	14	Occipital	Normal	FCD1c	L	II
TE	F	7	23	Parietal	L parietal FCD	FCD2b	L	I
MC	M	7	35	Insular	Normal	NA	L	I
SJ	M	17	12	Frontal	R prefrontal gliotic scar (AVM)	Gliosis	R >L	II
CA	F	8	14	Temporo-frontal	Anterior temporal necrosis	Gliosis	R	III
BF	M	11	45	Temporo-frontal	R Frontal FCD	FCD 2	R	I
FC	F	18	5	Temporo-parieto-opercular	Normal	Hippocampal sclerosis	R	IV
DM	F	8	13	Temporo-insular	R temporal anterior resection cavity	Gliosis	R	IV
CG	M	28	5	Temporal mesial	R temporo-polar & amygdala FCD, L post-chiasmal pilocytic astrocyrtoma	FCD 2b	R	III
MRA	M	40	4	Temporo-frontal	R fronto-temporal necrosis	Gliosis	R	I
MS	F	16	19	Temporal mesial	Hippocampal sclerosis	Hippocampal sclerosis	L	II
BC	F	24	17	Temporal mesial	Normal	NA	L	IV
SX	M	14	21	Motor-premotor	L insulo-opercular necrosis	NA	L	I

Supplementary Table 2: Region indices and labels in the Destrieux parcellation

Index	Short Name	Long Name
1	G_and_S_frontomargin	Fronto-marginal gyrus (of Wemicke) and sulcus
2	G_and_S_occipital_inf	Inferior occipital gyrus and sulcus
3	G_and_S_paracentral	Paracentral lobule and sulcus
4	G_and_S_subcentral	Subcentral gyrus (central operculum) and sulci
5	G_and_S_transv_frontopol	Trasverse frontopolar gyri and sulci
6	G_and_S_cingul-Ant	Anterior part of the cingulate gyrus and sulcus
7	G_and_S_cingul-Mid-Ant	Middle-anterior part of the cingulate gyrus and sulcus
8	G_and_S_cingul-Mid-Post	Middle-posterior part of the cingulate gyrus and sulcus
9	G_cingul-Post-dorsal	Posterior-dorsal part of the cingulate gyrus
10	G_cingul-Post-ventral	Posterior-ventral part of the cingulate gyrus
11	G_cuneus	Cuneus
12	G_front_inf-Opercular	Opercular part of the inferior frontal gyrus
13	G_front_inf-Orbital	Orbital part of the inferior frontal gyrus
14	G_front_inf-Triangul	Triangular part of the inferior frontal gyrus
15	G_front_middle	Middle frontal gyrus
16	G_front_sup	Superior frontal gyrus
17	G_Ins_lg_and_S_cent_ins	Long insular gyrus and central sulcus of the insula
18	G_insular_short	Short insular gyri
19	G_occipital_middle	Middle occipital gyrus
20	G_occipital_sup	Superior occipital gyrus
21	G_oc-temp_lat-fusifor	Lateral occipito-temporal gyrus (fusiform gyrus)
22	G_oc-temp_med-Lingual	Lingual gyrus, lingual part of the medial occipito-temporal gyrus
23	G_oc-temp_med-Parahip	Parahippocampal gyrus, parahippocampal part of the medial occipito-temporal gyrus
24	G_orbital	orbital gyri
25	G_pariet_inf-Angular	Angural gyrus
26	G_pariet_inf-Supramar	Supramarginal gyrus
27	G_parietal_sup	Superior parietal lobule
28	G_postcentral	Postcentral gyrus
29	G_precentral	Precentral gyrus
30	G_precuneus	Precuneus
31	G_rectus	Straight gyrus, Gyrus rectus
32	G_subcallosal	Subcallosal area, subcallosal gyrus
33	G_temp_sup-G_T_transv	Anterior transverse temporal gyrus
34	G_temp_sup-Lateral	Lateral aspect of the superior temporal gyrus
35	G_temp_sup-Plan_polar	Planum polare of the superior temporal gyrus
36	G_temp_sup-Plan_tempo	Planum temporale or temporal plane of the superior temporal gyrus
37	G_temporal_inf	Inferior temporal gyrus
38	G_temporal_middle	Middle temporal gyrus
39	Lat_Fis-ant-Horizont	Horizontal ramus of the anterior segment of the lateral sulcus (or fissure)
40	Lat_Fis-ant-Vertical	Vertical ramus of the anterior segment of the lateral sulcus (or fissure)

Supplementary Table 2: Region indices and labels in the Destrieux parcellation

**Index**	**Short Name**	**Long Name**
41	Lat_Fis-post	Posterior ramus of the lateral sulcus (or fissure)
42	Pole_occipital	Occipital pole
43	Pole_temporal	Temporal pole
44	S_calcarine	Calcarine sulcus
45	S_central	Central sulcus
46	S_cingul-Marginalis	Marginal branch of the cingulate sulcus
47	S_circular_insula_ant	Anterior segment of the cirucular sulcus of the insula
48	S_circular_insula_inf	Inferior segment of the cirucular sulcus of the insula
49	S_circular_insula_sup	Superior segment of the cirucular sulcus of the insula
50	S_collat_transv_ant	Anterior transverse collateral sulcus
51	S_collat_transv_post	Posterior transverse collateral sulcus
52	S_front_inf	Inferior frontal sulcus
53	S_front_middle	Middle frontal sulcus
54	S_front_sup	Superior frontal sulcus
55	S_interm_prim-Jensen	Sulcus intermedius primus (of Jensen)
56	S_intrapariet_and_P_trans	Intraparietal sulcus and transverse parietal sulcus
57	S_oc_middle_and_Lunatus	Middle occipital sulcus and lunatus sulcus
58	S_oc_sup_and_transversal	Superior occipital sulcus and transverse occipital sulcus
59	S_occipital_ant	Anterior occipital sulcus and preoccipital notch (temporo-occipital incisure)
60	S_oc-temp_lat	Lateral occipito-temporal sulcus
61	S_oc-temp_med_and_Lingual	Medial occipito-temporal sulcus (collateral sulcus) and lingual sulcus
62	S_orbital_lateral	Lateral orbital sulcus
63	S_orbital_med-olfact	Medial orbital sulcus (olfactory sulcus)
64	S_orbital-H_Shaped	Orbital sulci (H-shaped sulci)
65	S_parieto_occipital	Parieto-occipital sulcus (or fissure)
66	S_pericallosal	Pericallosal sulcus (S of corpus callosum)
67	S_postcentral	Postcentral sulcus
68	S_precentral-inf-part	Inferior part of the precentral sulcus
69	S_precentral-sup-part	Superior part of the precentral sulcus
70	S_suborbital	Suborbital sulcus
71	S_subparietal	Subparietal sulcus
72	S_temporal_inf	Inferior temporal sulcus
73	S_temporal_sup	superior temporal sulcus
74	S_temporal_transverse	Transverse temporal sulcus
