# Supplementary material for: Identifying spatio-temporal seizure propagation patterns in epilepsy using Bayesian inference
Source: Commun Biol. 2021 Nov 1;4:1244. doi: 10.1038/s42003-021-02751-5 (PMC8560929; doi:10.1038/s42003-021-02751-5)
Supplement: Supplementary file 2 — Reporting Summary [file 42003_2021_2751_MOESM2_ESM.pdf]

nature research

Corresponding author(s): Viktor Jirsa

Last updated by author(s): Sep 20, 2021

Reporting Summary

Nature Research wishes to improve the reproducibility of the work that we publish. This form provides structure for consistency and transparency in reporting. For further information on Nature Research policies, see our Editorial Policies and the Editorial Policy Checklist.

Statistics

For all statistical analyses, confirm that the following items are present in the figure legend, table legend, main text, or Methods section.

n/a	Confirmed	
☐	☒	The exact sample size (*n*) for each experimental group/condition, given as a discrete number and unit of measurement
☒	☐	A statement on whether measurements were taken from distinct samples or whether the same sample was measured repeatedly
☒	☐	The statistical test(s) used AND whether they are one- or two-sided<br>*Only common tests should be described solely by name; describe more complex techniques in the Methods section.*
☒	☐	A description of all covariates tested
☒	☐	A description of any assumptions or corrections, such as tests of normality and adjustment for multiple comparisons
☒	☐	A full description of the statistical parameters including central tendency (e.g. means) or other basic estimates (e.g. regression coefficient) AND variation (e.g. standard deviation) or associated estimates of uncertainty (e.g. confidence intervals)
☒	☐	For null hypothesis testing, the test statistic (e.g. *F*, *t*, *r*) with confidence intervals, effect sizes, degrees of freedom and *P* value noted<br>*Give P values as exact values whenever suitable.*
☐	☒	For Bayesian analysis, information on the choice of priors and Markov chain Monte Carlo settings
☒	☐	For hierarchical and complex designs, identification of the appropriate level for tests and full reporting of outcomes
☒	☐	Estimates of effect sizes (e.g. Cohen's *d*, Pearson's *r*), indicating how they were calculated

Our web collection on statistics for biologists contains articles on many of the points above.

Software and code

Policy information about availability of computer code

Data collection	MRI data is acquired using Siemens Magnetom Verio 3T MR-scanner. Stereotactic EEG data is collected using a 128 channel Deltamed System
Data analysis	Freesurfer is used in generating structural connectomes from diffusion MRI data. STAN is used for Bayesian inference.

For manuscripts utilizing custom algorithms or software that are central to the research but not yet described in published literature, software must be made available to editors and reviewers. We strongly encourage code deposition in a community repository (e.g. GitHub). See the Nature Research guidelines for submitting code & software for further information.

Data

Policy information about availability of data

All manuscripts must include a data availability statement. This statement should provide the following information, where applicable:

- Accession codes, unique identifiers, or web links for publicly available datasets
- A list of figures that have associated raw data
- A description of any restrictions on data availability

The patient data sets cannot be made publicly available due to the data protection concerns.

Field-specific reporting

Please select the one below that is the best fit for your research. If you are not sure, read the appropriate sections before making your selection.

☒ Life sciences ☐ Behavioural & social sciences ☐ Ecological, evolutionary & environmental sciences

For a reference copy of the document with all sections, see nature.com/documents/nr-reporting-summary-flat.pdf

Life sciences study design

All studies must disclose on these points even when the disclosure is negative.

Sample size	Data of 25 patients where surgical outcome is available have been selected for testing the accuracy of model predictions.
Data exclusions	No data is excluded
Replication	All analysis has been repeated multiple times with different parcellations and we confirm that the findings are reproducible
Randomization	Patients are divided into two groups based on the surgical outcome. One group contains patients where surgery achieved seizure freedom and the second group contains patients where post surgical seizure freedom is not achieved. More details of the patient groups are given in the Methods section of the manuscript
Blinding	Blinding is not relevant to our study as patients are grouped based on surgical outcomes i.e. post surgical seizure freedom versus recurring seizures.

Reporting for specific materials, systems and methods

We require information from authors about some types of materials, experimental systems and methods used in many studies. Here, indicate whether each material, system or method listed is relevant to your study. If you are not sure if a list item applies to your research, read the appropriate section before selecting a response.

Materials & experimental systems

n/a	Involved in the study	
☒	☐	Antibodies
☒	☐	Eukaryotic cell lines
☒	☐	Palaeontology and archaeology
☒	☐	Animals and other organisms
☐	☒	Human research participants
☒	☐	Clinical data
☒	☐	Dual use research of concern

Methods

n/a	Involved in the study	
☒	☐	ChIP-seq
☒	☐	Flow cytometry
☐	☒	MRI-based neuroimaging

Human research participants

Policy information about studies involving human research participants

Population characteristics	Empirical data used in this study consists of 25 patients with drug resistant epilepsy of which 15 are female and 10 are male. Epilepsy onset age ranged from 2 years to 55 years with a mean age of 18.84 and standard deviation of 12.63
Recruitment	Patients were selected as part of a normal clinical routine
Ethics oversight	Comiteé de Protection des Personnes Sud-Meéditerranée I

Note that full information on the approval of the study protocol must also be provided in the manuscript.

Magnetic resonance imaging

Experimental design

Design type	Not applicable. No funciontal MRI data is used in this study
Design specifications	Not applicable
Behavioral performance measures	Not applicable

Acquisition

Imaging type(s)	Diffusion MRI
Field strength	3 Tesla
Sequence & imaging parameters	DTI-MR sequence either with repetition time = 10.7 s, echo time = 95 ms, voxel size 1.95 x 1.95 x 2.0 mm or repetition time = 3 s, echo time = 88 ms, voxel size 2.0 x 2.0 x 2.0 mm
Area of acquisition	Whole brain

Diffusion MRI ☒ Used ☐ Not used

Parameters: DTI-MR sequence, either with angular gradient set of 64 directions, repetition time = 10.7 s, echo time = 95 ms, voxel size 1.95 x 1.95 x 2.0 mm, b-weighting of 1000 s/mm^2, or with angular gradient set of 200 directions, repetition time = 3 s, echo time = 88 ms, voxel size 2.0 x 2.0 x 2.0 mm, b-weighting of 1800 s/mm^2

Preprocessing

Preprocessing software	Freesurfer v6.0.0 is used for segmentation to Destrieux parcellation, FSL V6.0 is used for coregistration with 12 degrees of freedom, MRtrix v0.3.15 is used for tractography
Normalization	Not applicable. Preprocessing until the connectome estimation is on the subject level
Normalization template	Not applicable
Noise and artifact removal	Not applicable
Volume censoring	Not applicable

Statistical modeling & inference

Model type and settings	Not applicable
Effect(s) tested	Not applicable

Specify type of analysis: ☒ Whole brain ☐ ROI-based ☐ Both

Statistic type for inference (See Eklund et al. 2016)	Not applicable
Correction	Not applicable

Models & analysis

n/a	Involved in the study
☒	☐ Functional and/or effective connectivity
☒	☐ Graph analysis
☒	☐ Multivariate modeling or predictive analysis
